# MBE: model-based enrichment estimation and prediction for differential sequencing data

**DOI:** 10.1186/s13059-023-03058-w

**Published:** 2023-10-02

**Authors:** Akosua Busia, Jennifer Listgarten

**Affiliations:** Department of Electrical Engineering & Computer Science, University of California, Berkeley, Berkeley, 94720 CA USA

**Keywords:** Differential analysis, Machine learning, Selection experiments, Protein engineering, Sequencing

## Abstract

Characterizing differences in sequences between two conditions, such as with and without drug exposure, using high-throughput sequencing data is a prevalent problem involving quantifying changes in sequence abundances, and predicting such differences for unobserved sequences. A key shortcoming of current approaches is their extremely limited ability to share information across related but non-identical reads. Consequently, they cannot use sequencing data effectively, nor be directly applied in many settings of interest. We introduce model-based enrichment (MBE) to overcome this shortcoming. We evaluate MBE using both simulated and real data. Overall, MBE improves accuracy compared to current differential analysis methods.

**Supplementary information:**

The online version contains supplementary material available at 10.1186/s13059-023-03058-w.

## Background

Using next-generation sequencing, we can now assay up to billions of DNA or RNA sequences in parallel for an ever-expanding set of properties of interest [1–3]. As a consequence, high-throughput sequencing has dramatically changed the landscape of biological discovery—both for basic scientific inquiry into cellular transcriptomes [4] and protein behavior and evolution [3, 5], and in application areas spanning human disease and variant detection [5, 6], engineering anti-viral immunogens and therapeutics [3, 7, 8], drug and antibiotic resistance [3, 5], regulatory element engineering in synthetic biology [9] and beyond. Across many of these scientific areas, a key desired outcome from a high-throughput sequencing experiment is to quantify the change in relative abundance of a particular sequence between two conditions for a large number of distinct sequences. This type of quantification is often referred to as estimating the “log-enrichment” of a sequence between two conditions [2, 5, 7, 8, 10–12]. For example, log-enrichment estimation is performed in differential analyses of RNA-seq and ATAC-seq experiments [4, 13–15] to quantitatively compare gene- or transcript-level expression—based on sequencing counts—between, for instance, a “normal” control condition and a gene knockdown condition that corresponds to the control condition except that a particular gene has been knocked out [4]. In this case, the log-enrichment is a real-valued number that is meant to characterize how much each gene or transcript abundance differs between the two conditions—the larger the log-enrichment for a transcript, the more it is believed this transcript is relevant to explaining changes between the two conditions. Log-enrichment is also commonly used to analyze selection experiments, wherein one condition may be meant to select for more desirable genes or proteins. For instance, in protein engineering, log-enrichment is used to compare sequencing reads before and after one subjects a library of proteins to a selection for a desired property that one is trying to engineer [3, 7, 8, 16–20]. As one example, one may be interested in what population of proteins emerges from the starting “pre-selection library” after being subjected to a selection for catalytic activity [21]. We refer to the resulting selected proteins as the “post-selection library.” Other examples of high-throughput selections include selection for binding affinity to a specific target of interest [12, 22], enzymatic activity [21, 23], or infection of a specific cell type [7, 8, 20]. Indeed, such high-throughput selection experiments are frequently used for directed evolution [22, 24], deep mutational scanning [2, 3, 5, 10, 12, 25], and functional enrichment analysis [26], and have wide-ranging biologically significant applications, including antibody design [27, 28]; profiling pathogen proteomes for epitopes and major histocompatibility complex binding [17, 18]; improving thermostability [23]; assessing binding [12, 16, 22], catalytic activity [21, 23], and packaging efficiency or infectivity of viral vectors [7, 8, 19, 20].

By accurately estimating log-enrichment for large sequence libraries in these selection settings, one can identify sequences that are more (or less) likely to have desired properties, such as high affinity to the target in a high-throughput selection experiment for binding or a functional relationship to the knockdown gene in a differential RNA-seq experiment. Consequently, such estimates also have the potential to reveal insights into the sequence determinants of the property of interest. Increasingly, log-enrichment estimates are also being used as supervised labels for training machine learning models so that one may predict enrichment for unobserved sequences, or probe the model to gain further insights [16, 19, 20, 22, 29–32]. These supervised models are often more accurate than popular physics-based and unsupervised machine learning methods such as Rosetta and DeepSequence [32].

### Limitations of log-enrichment estimates

**Fig. 1:**
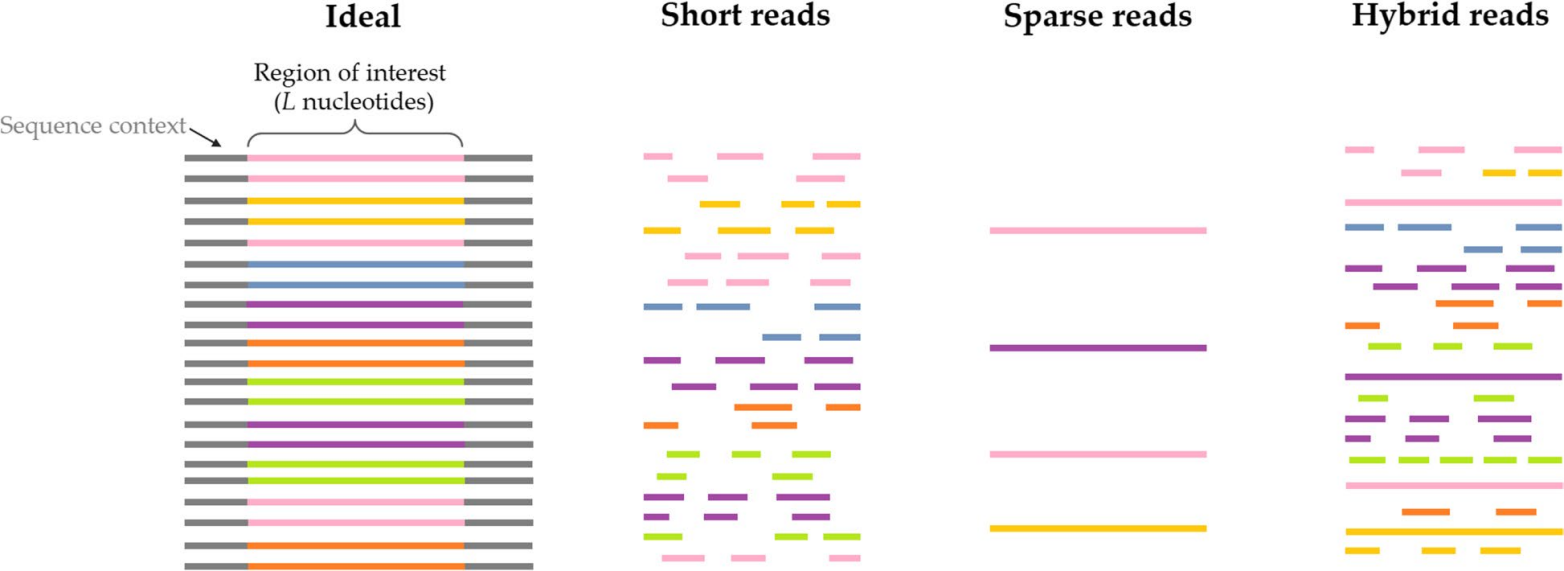
Illustration of sequencing settings that pose challenges for count-based log-enrichment approaches. “Ideal”: in an ideal world, we would always have high read coverage, with reads long enough to span the region of interest (length *L* ). “Short reads”: in the short read setting, short, possibly overlapping reads each only cover a portion of the region of interest. For illustrative purposes, we have displayed short reads in different positions and color-coded by sequence identity, but in general, positional information would not be known and would have to be deduced through read mapping or similar. “Sparse reads”: in the sparse read setting, even if the reads are sufficiently long to cover the region of interest, few are available, making cLE estimates high variance. “Hybrid reads”: in the hybrid read setting, long- and short-read datasets are combined into a single sequencing dataset. Consequently, this setting is plagued with problems that arise in both short and sparse settings, although the combined types of reads should in principle be useful

Although standard “count-based” log-enrichment (cLE) estimates calculated from observed read counts have proven incredibly useful, they suffer from one fundamental shortcoming that affects several practical settings. That shortcoming is the inability to share information across non-identical reads—reads that are non-identical either because they span different parts of a genomic sequence and/or they differ at one or more positions in a given span. Specifically, because cLE estimates require counting the number of times a *unique* genomic region is observed, cLE is only straightforwardly applicable when individual reads span the entire *region of interest*—i.e., the entire span of genomic sequence which we would like to quantify (Fig. 1). If instead, say, short reads only cover part of the region of interest, then to compute cLE, one must derive ad hoc methods to combine the short reads and then deduce some notion of an effective count for the combined reads. Consequently, the inability to naturally and coherently share information across related, yet non-identical reads causes a loss of important available information in a number of practical settings (Fig. 1), including:

1. *Short reads*: when short, possibly overlapping, reads are available that each only cover a portion of the region of interest. When each read is only a small span of this region, it is not clear how one should use such reads to obtain the counts necessary to compute cLE estimates for the region of interest without significant pre-processing and/or assumptions regarding the mapping between short reads and full-length sequences.
2. *Sparse reads*: when reads are sufficiently long to cover the region of interest, but so few sequencing reads are available that read counts are extremely low. This scenario commonly occurs with long-read sequencing [6, 33–35]. cLE estimates computed from such sparse (i.e., low read count) data are high variance [5, 11, 20]. However, many non-identical reads may contain shared information, for example, by containing identical motifs within reads that may otherwise differ in ways that are not relevant to the problem at hand (e.g., distinguishing the sequence determinants of viral packaging).
3. *Hybrid reads*: when a combination of long and short reads are collected and combined into a single sequencing dataset, as is sometimes done to combine the strengths of high-error nanopore-based sequencing and lower-error short-read sequencing [36]. In practice, estimation of cLE in the hybrid read setting is affected by both the short read and sparse read challenges just described, with the added heterogeneity of now having substantially different lengths in the same dataset. Although, in theory, the inclusion of long reads can help “sew” together the short reads, doing so requires careful modeling, the errors of which will cascade into the cLE estimates that rely on such a step to be able to count “reads” for the region of interest.
4. *Negative selection*: when the goal is to identify sequences enriched in a property that is opposite from the selection in the context of computing log-enrichment for a high-throughput selection experiment. This may occur when it is easy to develop an assay only for the opposite positive selection, and not for the desired negative selection. For example, in studies of adeno-associated virus (AAV) tropism [8, 37], one may wish to identify sequences that do not infect an off-target cell type when the only available high-throughput selection experiment selects *for* infecting the off-target cell type. A key desired outcome in the negative selection setting is to produce accurate log-enrichment estimates for sequences which do not tend to pass the selection (but occasionally do, so that we can count them), whereas typically one seeks to characterize those enriched for passing the selection. By design, the post-selection read counts for sequences of interest in negative selection are low, making their corresponding cLE estimates high variance.

For the case of sparse reads and negative selection (i.e., low sequencing counts), it is well-known that cLE estimates suffer from high variance  [5, 11, 20]. Previous efforts to reduce variance employ regression to “de-noise” cLE estimates by either using a model to intelligently aggregate data across iterative selection rounds [5] or by downweighting examples with low counts [16, 20]. While these techniques can yield higher-quality results, they are extremely limited in their ability to share information across non-identical reads. As a simple, intuitive example, if only 10 out of 300 positions in a sequence of interest are predictive of the property of interest, better statistical power could be achieved by calculating cLE using counts defined only by the 10 relevant positions rather than all 300, since the latter will cause most reads to appear to be non-identical and hence treated separately. A method that could automatically deduce such shared information, would be more powerful. This simple, intuitive scenario can be generalized well beyond this one example, as discussed next.

Ideally, to accurately estimate log-enrichment (LE), we would like sequencing data with high read coverage that is comprised of reads that each individually cover the full region of interest (Fig. 1). However, in practice, individual reads may not cover the entire region of interest—such as is likely to arise with short-read sequencing, but could also occur when using long-read technologies to analyze large sequences of interest [35]. In these settings, it is not obvious how to count reads for the region of interest, nor how to calculate the desired cLE estimates. Because the hybrid read setting contains short reads, it is also likely to be plagued by this issue. To tackle the LE estimation problem nonetheless, one might consider estimating cLE for shorter, sub-regions-of-interest (of length less than or equal to the typical read length) and then devising a heuristic to add these cLE estimates together to produce a LE estimate for the actual region of interest. However, it would be difficult for such an approach to correctly account for correlations between reads (e.g., owing to linkage disequilibrium), and to allow for data sharing by way of partial overlap between reads. Moreover, of all the possible heuristics one might consider to do such an analysis, is not clear which to use, and the answer likely depends on the specific application. If we could bypass such a modeling step, and instead directly estimate LE, we could stand to benefit substantially.

In applications where there is a known reference sequence—such as in many RNA-seq and ATAC-seq experiments—the reference can help provide information about how to combine reads [4]. However, this is typically performed by alignment and assembly, followed by cLE estimation; thus such approaches also suffer from many of the same limitations just described. Devising an alternative approach to LE estimation—one that is capable of both “sewing” together partially overlapping reads and, more generally, sharing across non-identical reads—would enable more efficient sharing of information.

### A new approach for log-enrichment estimation

Ultimately, a method that can automatically learn to share information as appropriate across non-identical reads will improve our ability to extract important information from sequencing data in a range of settings. Herein, we propose and evaluate a novel, coherent framework that enables us to do just that. Our manuscript is organized as follows: next, we (i) detail how log-enrichment estimates are currently computed; (ii) provide a high-level overview of our approach, *model-based enrichment* (MBE); (iii) provide a detailed empirical characterization of MBE using data from simulated high-throughput selection experiments; and (iv) do the same on real experimental data.

Overall, we find empirically that MBE enables effective analysis across a broader range of common experimental setups in use today, including when short-read, long-read, or both types of sequencing reads are used. Our primary motivation is to improve *predictions* of log-enrichment on new (unobserved) sequences, as this is most relevant to our own work in machine learning-guided library design. However, our results show that MBE also enables better *estimation* of log-enrichment, the more classical use case. We show that, compared to existing approaches based on cLE, over a broad range of settings, MBE produces predictions that correlate better with true labels. We show that this is, in part, a downstream consequence of the fact that MBE is more robust to low sequencing counts. We also show that MBE enables better characterization of sequences of interest from a negative selection experiment. Additionally, we find MBE performs better at selectivity experiments, wherein two selection experiments are performed and one wants to identify sequences that are highly selected for in one (the positive selection), and highly selected against in the other (negative selection)—such as we might seek when designing gene therapy viral vectors to infect one cell type and not another. This setting thus requires modeling of three conditions, instead of the typical two we have in other experiments.

## Results

### Overview of current log-enrichment estimation approaches

Given sequencing read counts from two libraries corresponding to two conditions, *A* and *B*, cLE estimates are typically calculated by: (i) assigning an index, *i*, to each unique sequence in the sequencing data from libraries *A* and *B*, denoting each by $x_i$; (ii) computing read counts, $n_i^A$ and $n_i^B$, specifying the number of times $x_i$ appeared in the sequencing data from each library; (iii) normalizing the counts $n_i^A$ and $n_i^B$ by the total number of reads from each library, $N^A$ and $N^B$; (iv) and, finally, taking the log-ratio of the normalized counts. Thus, the cLE estimate, $\log e_i$, is given by $\log e_i = \log \frac{n_i^B / N^B}{n_i^A /N^A }$. The estimate $\log e_i$ has higher variance when the counts $n_i^A$ and $n_i^B$ are lower. For example, for fixed $N^A$ and $N^B$, a sequence with $n_i^A = 1$ and $n_i^B = 2$ has the same $\log e_i$ as a sequence with $n_i^A = 100$ and $n_i^B = 200$, yet the latter is supported by 100 times more evidence (and thus is a lower-variance estimate).

Supervised machine learning regression models have been used to reduce the variance of (i.e., “de-noise”) such cLE estimates [5, 16, 20], and to make predictions for sequences not present in the training library [19, 20, 22, 32]. The latter strategies, which we refer to as *LE regression* approaches, use cLE estimates as supervised labels to learn a predictive model mapping from sequence to predicted LE. Zhu et al. [20] additionally derive a variance estimate for $\log e_i$ which enables them to weight each training sequence according to the amount of evidence that supports its cLE estimate, yielding improved predictive performance. Consequently, when comparing to a baseline for LE prediction, we use this same approach, which we refer to as *weighted LE regression* (wLER).

### A new approach: model-based enrichment

Existing approaches that use regression-based LE estimation (or prediction) are performed in two sequential steps: first, compute a cLE estimate for each unique sequence [2, 5, 10, 11], and second, train a regression model to predict these cLE estimates from the observed sequences, possibly weighting each sequence to account for its corresponding level of evidence [20, 32]. In contrast, our newly introduced method, MBE, performs both of these steps at once, resulting in a more powerful and more general analysis framework. We do so by reframing the LE estimation problem: we show that a cLE estimate can be viewed as a sample-based estimate of the logarithm of what is known as a *density ratio*—the ratio of probability densities of the observed sequences under each condition. Therefore, we can estimate and predict LE by solving a *density ratio estimation* (*DRE*) problem. Furthermore, it has been shown that DRE can be effectively and accurately performed by training a probabilistic classifier to predict which of the two densities a sample came from (e.g., condition *A* or *B*) [38–44]. Specifically, the ratio of such a classifier’s predicted class probabilities provably converges to the density ratio [39–41].

**Fig. 2:**
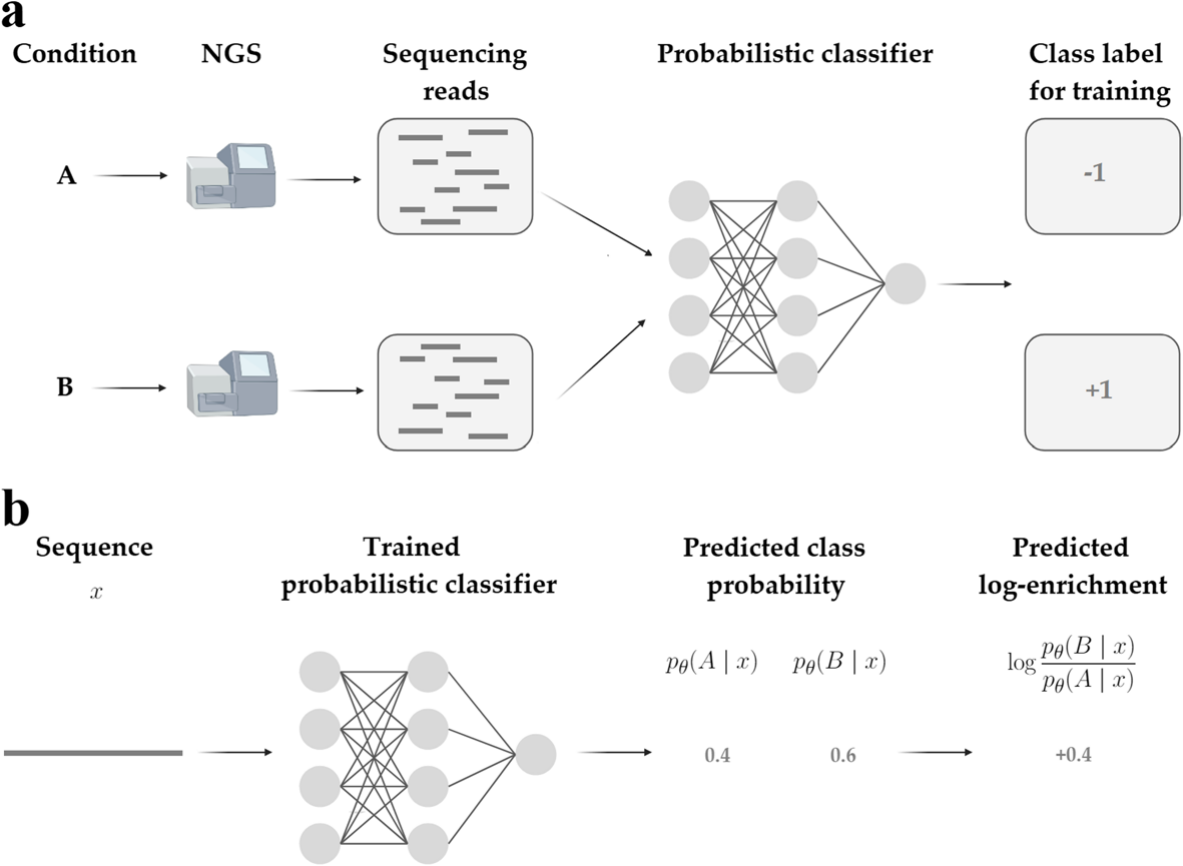
Overview of MBE at train time and prediction time. **a** At train time, next-generation sequencing reads from each condition, A and B, are used to train a probabilistic classifier. Without loss of generality, we encode condition A using the class label $-1$ and condition B using the class label $+1$ to train the classifier. More specifically, when applying MBE to the simulated data, we generate *N* training reads for condition A, and *N* training reads for condition B (i.e., equal class sizes). Then, we train a classifier on the $N+N=2N$ data points, where each data point is a one-hot encoding of one read sequence with a corresponding label ($-1$ for class A or $+1$ for class B). (For all neural network models, we used a one-hot encoding. For linear models, we used other feature sets—see the “Methods” section. One could add any other features as desired, such as a mapped genomic position). The same read can appear more than once and may appear with discordant labels (e.g., if it appears in both conditions). The only difference in the implementation on the real data as compared to the simulated, is that the total number of reads may be quite different between the different conditions; to account for this, we re-weight the classifier loss function by the total number of reads in each condition to equalize the impact of each condition on the classifier (see the “Methods” section). **b** At prediction time, a single sequence is given to the trained classifier, which produces a predicted probability for each condition (class). For a two-condition model (A *vs.* B), we then compute the logarithm of the ratio of the two class probabilities to obtain the LE. When more than two conditions (classes) are used, a LE can be computed for any pair of conditions using the same calculations. For example, for a three-condition (three-class) model, there are $$\left( {\begin{array}{c}3\\ 2\end{array}}\right) = 3$$ possible LEs that can be computed, each between two of the conditions

Through this series of theoretically justified steps, we are able to transform the problem of estimating LE into one of training a read-level classifier to distinguish which condition a read came from (Fig. 2). This transformation provides several distinct advantages over existing methods, which we outline here and, later demonstrate empirically. One advantage is that, unlike regression-based approaches like wLER, MBE does not rely on cLE estimates as labels, which as discussed earlier, are affected by a fundamental shortcoming. Instead, MBE bypasses this step altogether by training a classifier directly on the raw sequence data with accurate labels corresponding to the known condition the read came from. Consequently, because MBE is classifier-based, it is easy to implement using standard software packages for training and tuning machine learning classifiers: one need simply train a classifier using any standard classification tools, and can perform model selection using standard cross-validation techniques. In particular, MBE can readily make use of modern-day neural network models in a plug-and-play manner, which also enables us to easily handle (possibly overlapping) reads of different lengths. For example, fully convolutional neural network classifiers naturally handle variable-length sequences because the convolutional kernels and pooling operations in each layer are applied in the same manner across the input sequence, regardless of its length. Similarly, the same can be said for transformer architectures. A further advantage is that our approach naturally accounts for differing levels of evidence per sequence of interest, which in previous LE regression methods was either ignored or addressed post hoc under specific distributional assumptions [16, 20]. Finally, our approach trivially generalizes to settings with more than two conditions of interest by replacing the binary classifier with a multi-class classifier. The multi-class classification model is trained to predict the condition from which each read arose; then, the density ratio for any pair of conditions can be estimated using the ratio of its corresponding predicted class probabilities (see the “Methods” section). This generalization enables us to naturally handle experiments with multiple properties of interest, such as our simulated sequence selectivity experiments.

We highlight that our classifier-based DRE approach differs substantially from several recent approaches that also make use of classification. In one, cLE estimates are thresholded and a classifier built to predict the resulting binarized labels (e.g., [19]). In another, a classifier is built to predict whether a sequence appeared at all in post-selection sequencing data (e.g., [30]). Neither of these approaches address the shortcomings that we seek to resolve with MBE.

#### Technical overview of MBE

Here, we provide more detail about our MBE approach (see the “Methods” section for full detail). Recall that the cLE estimate is the log-ratio of the two normalized counts, $\frac{n_i^A}{N^A} \text { and } \frac{n_i^B}{N^B}$. These normalized counts are also the empirical frequencies of the $i^\text {th}$ unique read, $x_i$, in the sequencing data for conditions *A* and *B*, respectively. In particular, these two ratios are the sample-based estimates of the population frequencies of $x_i$ in each library. We denote the population frequencies by the probabilities $p^A(x_i)$ and $p^B(x_i)$. Consequently, $\log e_i$ can be viewed as a sample-based estimate of the population-level LE, which we denote $\log d(x_i)$. Specifically, $\log e_i \approx \log d(x_i) = \log \frac{p^B(x_i)}{p^A(x_i)}$, where *d* is the *density ratio* between the library distributions. By training a binary classifier with parameters, $\theta$, to predict the probability that a read with sequence $x_i$ came from library *B*, $p_\theta (l=B \mid x_i)$, we can estimate $\log d(x_i)$, and hence the LE, as $\log d(x_i) \approx \log \frac{p_\theta (l=B \mid x_i)}{1-p_\theta (l=B \mid x_i)}$ [39, 41] (Fig. 2). It has been proven theoretically that under a correctly specified model, this density ratio estimation method is optimal among a broad class of semi-parametric estimators—that includes the wLER method—in terms of asymptotic variance [39] (Additional file 1: Supplementary Note 1).

#### Contrasting wLER and MBE

At first glance, it may appear that the main difference between wLER and MBE is that the former uses regression, while the latter uses classification. However, conceptually, the more important difference is that wLER (and any regression-based LE method) uses cLE estimates as regression labels. Hence, wLER is dependent on cLE estimation and consequently is affected by all the limitations of cLE described earlier—namely those arising from the inability to share information across similar, but distinct reads. In contrast, MBE is trained using the raw reads directly, labeling each as coming from one of two conditions, thereby bypassing the issues arising from cLE estimates. That is, MBE’s classification labels need not be estimated, but are given to us for free, with 100% accuracy. It is this distinction that lies at the core of why MBE tends to outperform wLER. That one method uses classification instead of regression is a byproduct of the technical underpinnings used to achieve this *desideratum*.

#### Generalization of wLER and MBE to more than two conditions

Both wLER and MBE are straightforward to generalize to the setting of having more than two conditions, where one might be interested in estimating or predicting LE between any or all pairs of conditions among several conditions (such as arises when identifying selective sequences—see *Selection for sequence selectivity*). For wLER, one simply uses cLE to estimate regression labels for any pair of interest, and then trains a multi-output regression model—with one output for each such pair—using wLER with these labels and sequences (see the “Methods” section). For MBE, one simply augments the classification labels to have as many unique class labels are there are conditions—such as $\{-1,0,1\}$ for three conditions—and trains a multi-class classifier. Multi-class classification is a standard machine learning problem that typically employs a generalized logistic loss called a softmax loss. Concretely, the multi-class classifier yields a model of the probability $p_\theta (l=j \mid x_i)$, where the condition is indexed by *j*. The trained multi-class classifier can be used to estimate the density ratios between two conditions *j* and $j'$ using $\log d(x_i) \approx \log \frac{p_\theta (l=j \mid x_i)}{p_\theta (l=j' \mid x_i)}$. Note that this is particularly convenient because it scales linearly in the number of conditions. One potential, but unexplored, benefit of this is that one could easily compute LEs between two conditions where one is an aggregate of the original conditions. For example, suppose we used MBE to analyze three conditions, A, B, and C. Then MBE could be used to compute LEs between all three pairs of conditions (A *vs.* B, B *vs.* C, and A *vs.* C), but it could also be used to compare, for example, condition A to conditions B and C *jointly* using the same underlying classification model. To do so, one would simply compute $\log \frac{p_\theta (l=A \mid x_i)}{p_\theta (l=B \mid x_i) + p_\theta (l=C \mid x_i)}$. In contrast, the multi-condition generalization of wLER requires deciding which pairs of conditions are of interest before training the model and then computing labels for all such pairs separately.

### Overview of simulation experiments

**Fig. 3:**
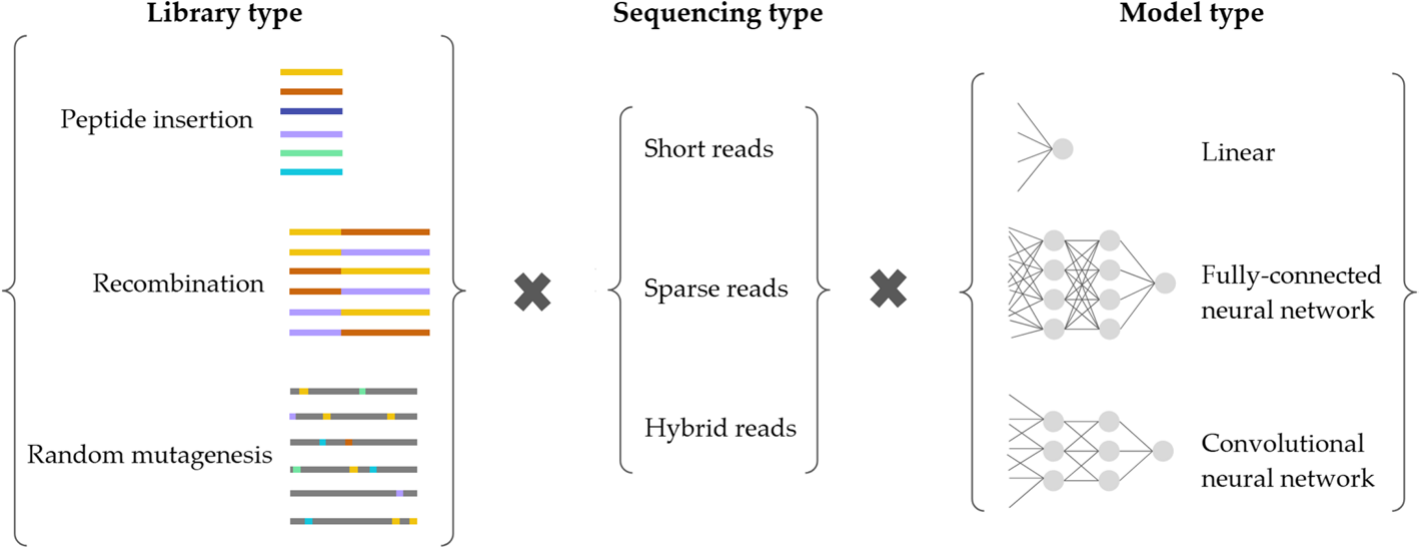
Overview of experiments on simulated datasets. We simulated three types of protein libraries: (i) peptide insertion, (ii) AAV recombination, and (iii) avGFP random mutagenesis libraries (Table 1). We simulated three types of sequencing datasets: (i) short reads (for all libraries), (ii) long reads (for all libraries except the peptide insertion libraries, where short reads cover the entire region of interest), and (iii) hybrid reads (for the AAV recombination library). We employed three types of architectures: (i) linear, (ii) fully-connected neural network, and (iii) convolutional neural network—using a classification head for MBE and a regression head for wLER. For short-read and hybrid-read datasets, we only used convolutional neural networks because only they can operate on variable-length sequences

Next, we describe the simulation experiments used to empirically compare and contrast our MBE approach with cLE and wLER across a broad range of settings (Fig. 3). We provide an overview of the simulated datasets, model architectures, and evaluation metrics, before presenting the results.

#### Simulated data

We sought to understand the strengths and weaknesses of the MBE and wLER approaches as we changed the following simulation settings:

1. The length of the sequence of interest, *L*, ranging from 21 to 2253 nucleotides.
2. Whether short or long reads were used (300 *vs.* 10,000 nucleotides).
3. The number of unique sequences in the theoretical pre- and post-selection libraries, $M'$, ranging from $8.5 \times 10^6$ to $2.6 \times 10^7$.
4. The number of pre- and post-selection reads, $N^\text {pre}$ and $N^\text {post}$—always set equal to each other, ranging from $4.6 \times 10^3$ to $4.6 \times 10^7$.
5. The complexity of the functional mapping between sequence and property of interest; this complexity was characterized in terms of a summary parameter controlling the amount of epistasis, *T*.

We simulated libraries that correspond to three types of experimental library constructions:

- *Insertion* of a sequence into a fixed background. In a given library, the insertion has fixed-length and a fixed position within the background sequence. The insertion library construction is motivated by our work in adeno-associated virus (AAV) capsid engineering which aims to understand sequence determinants of AAV properties such as packaging [20]. In this study, the sequence of interest is a 21-mer nucleotide insertion sequence into the capsid with fixed background. Herein, we simulate this insertion library with varying lengths (21, 150, and 300 nucleotides). The pre-selection library is generated to be roughly uniform in nucleotide space (technically, an “NNK” degenerate codon distribution).
- *Random mutagenesis*—motivated by a study to understand the fitness landscape of a green fluorescent protein of length 714 nucleotides [31]. Herein, we mutagenize the green fluorescent protein across all positions using a 10% mutation rate to generate the pre-selection library.
- *Recombination*—motivated by an AAV directed evolution study [8], wherein several AAV serotypes are recombined using seven crossovers separating eight recombination blocks. Herein, we generate library sequences by recombining nine AAV serotypes using eight equally sized blocks. The total length of all eight blocks is 2253 nucleotides.

A summary of the simulated sequencing datasets is provided in Table 1.

**Table 1:** Summary of simulated datasets. For each dataset, we list the library name (Library), sequence length in nucleotides (*L*), number of unique library sequences ($M'$), epistasis hyperparameter used for fitness simulation (*T*), read type (short, long, or hybrid), $\%$ of the sequence of interest covered by individual reads (Cover), and number of pre-selection and post-selection reads ($N^\text {pre}$ and $N^\text {post}$), which were always equal. We simulate $4.6 \times 10^7$ short reads to match the experimental data from Zhu et al. [20], and up to $4.6 \times 10^5$ long reads to be within the current throughput of PacBio’s technologies [34, 35]. Each dataset is described in more detail in the Methods

Library	*L*	*M′*	*T*	Read type	Cover	$N^\text {pre}=N^\text {post}$
21-mer insertion	21	$8.5 \times 10^6$	140	Short	100	$4.6 \times 10^7$
150-mer insertion	150	$8.5 \times 10^6$	1000	Short	100	$4.6 \times 10^7$
300-mer insertion	300	$8.5 \times 10^6$	2000	Short	100	$4.6 \times 10^7$
avGFP mutagenesis	714	$2.5 \times 10^7$	4760	Long	100	$4.6 \times 10^5$
avGFP mutagenesis	714	$2.5 \times 10^7$	4760	Short	42	$4.6 \times 10^7$
AAV recombination	2253	$2.6 \times 10^{7}$	15,020	Long	100	$4.6 \times 10^5$
AAV recombination	2253	$2.6 \times 10^{7}$	15,020	Long	100	$4.6 \times 10^4$
AAV recombination	2253	$2.6 \times 10^{7}$	15,020	Long	100	$4.6 \times 10^3$
AAV recombination	2253	$2.6 \times 10^{7}$	15,020	Short	13	$4.6 \times 10^7$
AAV recombination	2253	$2.6 \times 10^{7}$	15,020	Hybrid	100 long + 13 short	$4.6 \times 10^3$ long + $4.5 \times 10^7$ short

Underlying each of the motivating selection experiments is a property on which the sequences get selected, such as protein fluorescence. To simulate selection, we must simulate the ground truth *fitness function* that maps sequence to property. We did so as a linear function of a number of features, including all independent amino acid sites, and *T* higher-order epistatic features drawn randomly from all possible such effects, in a manner that re-capitulates the distribution of these effects in a real protein fitness landscape. In particular, combining insights from several papers [45–47], we assumed that *T* scaled linearly with the length of the sequence of interest, with a fixed coefficient based on Poelwijk et al. [29].

Finally, the process to simulate reads from the pre- and post-selection libraries can be summarized as follows: first, we generate library sequences using one of the three previously described library construction simulations. Then, we randomly perturb the empirical distribution of the simulated library sequences (which simulates slight distributional perturbations that may occur with PCR amplification) to generate a pre-selection probability distribution. Next, the corresponding post-selection probability distribution is determined by scaling the pre-selection distribution according to the simulated fitness of the library sequences. Then, we sample reads that cover the full sequence of interest from the pre- and post-selection distributions. When simulating short reads, we truncate each of these reads to 300 nucleotides, at a position chosen uniformly at random. To be able to compare to the negative binomial modeling approach DEseq2 [14, 15, 48] used in the RNA-seq community, which requires multiple biological replicates, we repeated this read simulation process three times to generate three replicates (Additional file 1: Fig. S4). In all other results presented, no replicates were used, as this most closely mimics our typical use cases, including the real experimental datasets.

We also perform negative selection simulations, which were motivated by experiments wherein one seeks to identify sequences with a property, such as low-binding affinity, for which the only available assay enriches for the opposite, such as high-binding. Recall that this situation arises, for example, in studies of AAV tropism [8, 37] where the ideal viral vector selectively infects one cell type, but not others. We, therefore, aimed to estimate the accuracy of wLER and MBE to negatively select against an undesirable fitness. Moreover, we aimed to compare the methods’ abilities to accurately identify sequences of interest that are selective—meaning that they are simultaneously high in one fitness (the *positive fitness*) and low in a second (the *negative fitness*). To do so, we simulated two independent fitness functions and used each, separately, on the same pre-selection library to simulate two post-selection libraries and corresponding reads. Thus, these sequence selectivity experiments depend on being able to handle three conditions: the pre-selection, the post-selection for the positive fitness, and the post-selection for the negative fitness.

Although most of our simulations did not include sequencing errors, we constructed versions of two of the aforementioned datasets that did. For one of the insertion datasets, we used a uniform random substitution error rate of 0.1%, consistent with observed error rates of Illumina’s next-generation sequencers [49]. For one of the recombination datasets, we used SimLoRD [50] to simulate PacBio SMRT sequencing errors. As shall be seen, the noise had little effect on our results.

#### Model architectures

We implemented wLER and MBE using several model architectures. To enable direct comparison of the two methods, we kept the set of allowed architectures and hyper-parameters the same for both approaches, excluding the final layer and loss which dictate whether the model is for regression (wLER) or classification (MBE). Specifically, we used the seven model architectures in Zhu et al. [20]—three linear models and four fully-connected neural networks (NNs)—as well as four additional convolutional neural network (CNN) architectures. As the linear and NN architectures and hyper-parameters are from a paper that used wLER, to the extent the selected architectures may favor one of the approaches compared herein, they would favor wLER. The CNNs can operate on variable-length sequences, allowing us to train on short reads and make predictions on full-length sequences of interest. To provide a general comparison of the methods’ performance and robustness, we restrict our attention to this fixed set of model architectures and hyper-parameters, but in practice, for both MBE and wLER one can perform model and hyper-parameter selection on one’s specific dataset of interest using standard cross-validation.

Our selectivity experiments simulate selection for sequences that are simultaneously high in a desirable positive fitness and low in an undesirable negative fitness. For simplicity, in these experiments we allowed only one model architecture—the smallest NN architecture—to model the multiple conditions involved in these simulations. For MBE, this corresponds to using the NN as a three-class classification model—with one class corresponding to each of pre-selection, post-selection for the positive fitness, and post-selection for the negative fitness. For wLER, we used a two-output NN regression model with one output for the positive fitness and one for the negative. We used this architecture because it was the simplest non-linear model architecture we explored—meaning it is capable of capturing higher-order epistasis while being relatively parsimonious. Based on the results of our initial simulation experiments, this choice of architecture does not systematically benefit either of the wLER or MBE approaches (Additional file 1: Fig. S1).

#### Evaluation methods

We focused our comparisons on three competing approaches: standard cLE, wLER (recall this is a weighted regression on cLE), and our MBE approach which bypasses computation of cLE. wLER and MBE can both (i) make predictions on sequences not seen in the training data, and (ii) make model predictions on the training data itself to yield LE estimates—a sort of “de-noising” of the cLE estimates. We refer to these two tasks, respectively, as *prediction* and *estimation*. The cLE approach can only be used for estimation, hence it does not appear in prediction experiments. In the Supplementary Information, we also compared to the negative binomial-based estimation method, DEseq2 [48], which cannot be used for prediction and, unlike the other methods, requires multiple replicates.

To compare wLER to MBE on any given dataset, we used all model architectures and hyper-parameters for both methods, and then selected the best setting separately for each of wLER and MBE. No model or hyper-parameter selection is required for cLE since it does not use any model or have any parameters.

An important point to appreciate throughout our work is that although can use standard cross-validation on the sequencing data to pick hyper-parameters, we cannot do so to assess accuracy because this requires access to a true biophysical assay not plagued by the requirement to estimate LE. Hence, in simulated settings, we perform modified cross-validation wherein we evaluate performance on each fold by comparing predictions to the sequences’ ground truth fitness values.

We used threefold cross-validation to compute the Spearman correlation between ground truth fitness and predicted LE to compare and contrast the performance of each method. In our simulations, we can compare directly against ground truth LE values, and thus we also evaluate using Pearson correlation (which assumes a linear relationship between predicted and ground truth values), and mean squared error (MSE) which assumes shared units between predictions and ground truth metrics. However, we primarily focus on Spearman correlation because the other metrics are not appropriate when working with experimental data where, in general, one can expect LE and experimental property measurements (which are typically direct biophysical measures) only to have the same rank order but not necessarily a linear relationship. Additionally, we make use of a generalized Spearman correlation that focuses on sequences that have the highest ground truth LE—the focusing is controlled by a threshold on true LE which we sweep through a range of values, such that at one extreme, we compute the Spearman of all sequences in the test set, and on the other, of only the most truly enriched sequences (similarly to Zhu et al. [20]). The test set is always comprised of full sequences of interest, even when the training data contained reads that were shorter. For all cross-validation experiments, we averaged the Spearman correlations computed on each fold to produce one cross-validated correlation value. We use William’s t-test to assess statistical significance of the difference between the cross-validated Spearman correlations.

Each selectivity simulation is defined by two different simulated fitnesses, a positive fitness and negative fitness. We learn two-output models (one output per fitness) on these data. We define the selectivity of a sequence as the difference between its positive and negative fitness values. We apply the generalized Spearman correlation evaluation method described above for the positive fitness. For the negative fitness, we use a similar generalized Spearman correlation that focuses on sequences with *lowest*—instead of highest—ground truth LE. In the selectivity experiments, we also seek to compare how well wLER and MBE identify test sequences with high selectivity. To do so, for each method, we rank the sequences in each test fold according to predicted selectivity—the difference between predictions for each fitness—and take the top ten test sequences. Then, we compare the two ground truth fitness values of each of the chosen sequences to the fitness values of a theoretical optimally selective sequence that has the maximum true positive fitness and minimum true negative fitness observed in the given dataset. We also use McNemar’s test to assess the statistical significance of the difference between the methods’ accuracy at identifying the $1\%$ of test sequences with highest selectivity.

#### Results on simulated data

**Fig. 4:**
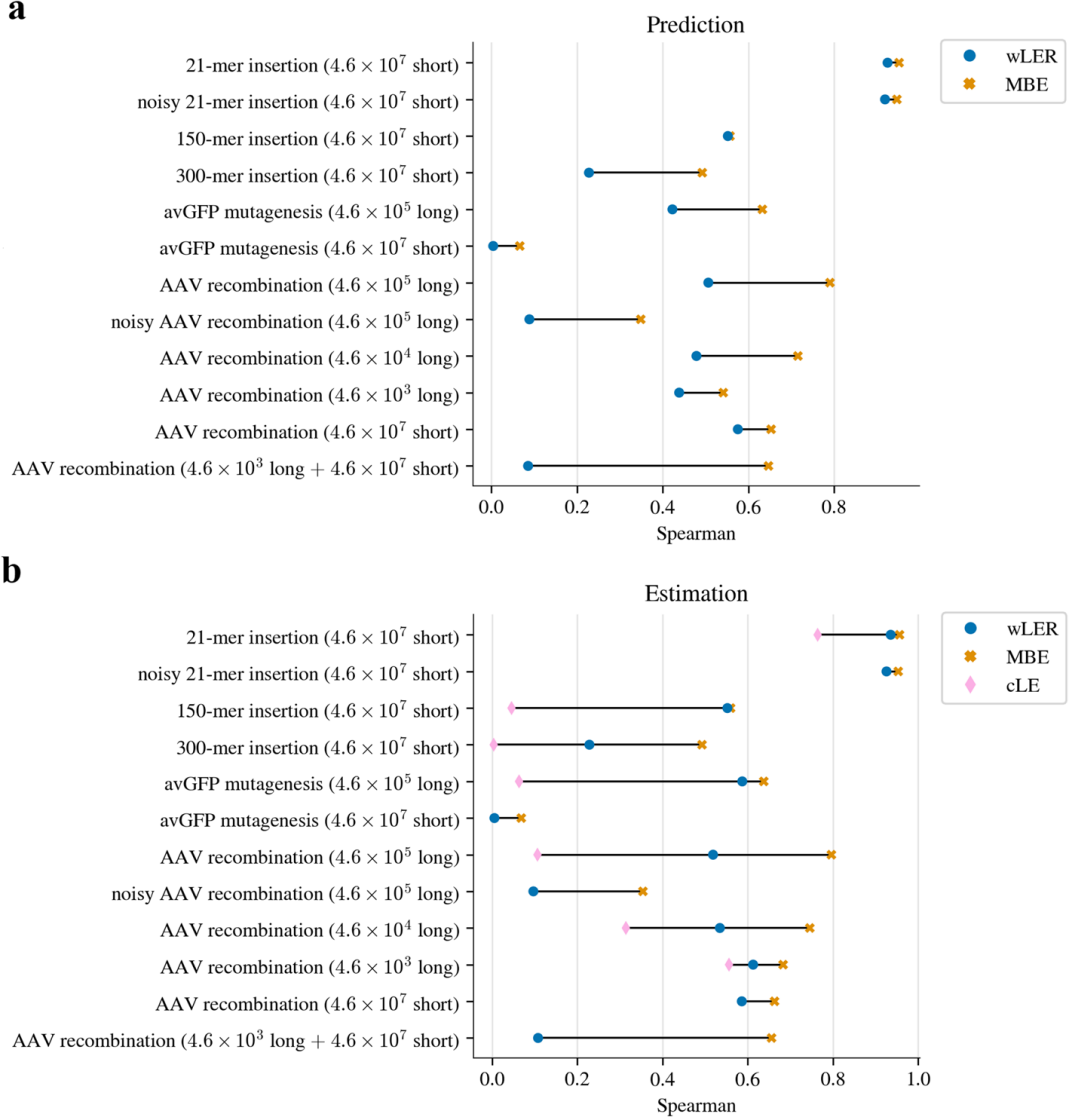
Simulated library results. Spearman correlation between ground truth fitness and cLE, wLER, and MBE estimates on full-length sequences of interest for the tasks of **a** prediction and **b** estimation. The cLE approach can only be used for estimation (not prediction), and additionally, only for experiments where the sequencing reads were long enough to cover the sequences of interest (“Cover" = 100). Thus, cLE is absent from some experiments. All differences are statistically significant ($p < 10^{-10}$). For wLER and MBE, the results shown are for the cross-validation-selected architecture for each approach, as described in the main text, and are presented in the order of Table 1. For comprehensive results across all model architectures see Additional file 1: Fig. S1. For comparisons using Pearson correlation and MSE see Additional file 1: Figs. S2–S3, and see Additional file 1: Fig. S4 for comparisons to DEseq2

Across all simulated datasets, our MBE approach made significantly more accurate LE predictions than wLER (Fig. 4a) according to standard Spearman correlation ($p < 10^{-10}$). The improvements of MBE over wLER in terms of Spearman correlation values were as much as 0.561 and as little as 0.005, with an average of 0.177. In no cases did MBE do worse than wLER. We also found that our MBE method performed better when faced with both Illumina- and PacBio-like sequencing error (Fig. 4, Additional file 1: Fig. S8). These performance benefits of MBE over wLER were not sensitive to the specific evaluation metric, and remained consistent using both Pearson correlation (Additional file 1: Fig. S2a) and MSE (Additional file 1: Fig. S3a). Moreover, MBE was much less sensitive to the choice of model architecture, to such an extent that even the worst-performing MBE model performed better than the best-performing wLER model on several datasets (Additional file 1: Fig. S1a). Similarly, for the estimation task, MBE outperformed wLER across all simulated datasets and evaluation metrics (Fig. 4b, Additional file 1: Figs. S2b, S3b, and S1b). We also found that MBE consistently improved estimation accuracy compared to the LE estimates produced using cLE and DEseq2 (Fig. 4b, Additional file 1: Fig. S4), although this result may depend on the specific type of replicates generated.

Collectively, our results demonstrate a clear win for MBE across a broad range of settings. In the subsequent sections, we examine the following specific settings to get a broader view of the strengths and weaknesses of each method: sparse reads, overlapping short-reads, hybrid long- and short-reads, negative selection, and selection for sequence selectivity.

##### Sparse read setting

We define the sparse read setting as occurring when the average number of sequencing reads per library sequence was lower than 0.02. In our experiments, this includes simulated long-read datasets for the avGFP mutagenesis and AAV recombination libraries. We hypothesized that the MBE approach would have a particular advantage in this setting because of its improved ability to combine information across similar but non-identical reads compared to cLE and wLER, the latter of which is trained using regression labels obtained from cLE estimates. On the prediction task, MBE maintains comparable accuracy to wLER on test sequences with high ground truth fitness, while improving accuracy in the other regimes (Additional file 1: Figs. S5a–b and S6). Additionally, MBE had lower variance than wLER across the different test folds (Additional file 1: Fig. S6). We also note that the longer the sequence of interest, the more MBE outperforms wLER—this nicely matches our intuition as the longer the read, the more sparse the setting (Fig. 4a, Additional file 1: Figs. S6d–e and S7). We observed similar trends for the estimation task (Fig. 4b, Additional file 1: Figs. S1b and S9). When we increase the total number of long reads for the AAV recombination library (from $4.6 \times 10^3$ to $4.6 \times 10^5$), more unique sequences with low counts occur in the data (Additional file 1: Fig. S7). Consequently, wLER is particularly challenged because it is trained using cLE estimates that cannot share data across non-identical reads to mitigate the effects of low counts. In fact, wLER is so challenged that, for many model architectures, its performance degrades when provided with more long-read sequencing data (Additional file 1: Fig. S5a–c). In contrast, MBE follows a more intuitive pattern: more training data always either maintained or improved performance, but never hurt the overall performance metrics (Fig. 4, Additional file 1: Fig. S5).

##### Short- and hybrid-read settings

In practice, experimenters often offset the sparsity of long-read sequencing by augmenting with higher-throughput short-read sequencing. We refer to this as the hybrid-read setting. Again, our results follow our intuition: for short-read and hybrid datasets, MBE outperformed wLER (Fig. 4a, Additional file 1: Fig. S5d–f). In fact, because wLER is trained using cLE estimates as its regression labels, it cannot leverage partial overlap between reads; consequently, its accuracy actually decreased when long-read data was supplemented with additional short reads, despite the fact that this creates a larger overall training set. Recall our hypothesis that, by avoiding the need to pre-compute and train on cLE estimates, the MBE approach is capable of learning to combine information across partially overlapping reads to make more efficient use of sequencing data. Indeed, our results support this hypothesis: MBE had higher accuracy when given a larger hybrid dataset, in contrast to wLER, which was less accurate with more training data.

##### Negative selection

**Fig. 5:**
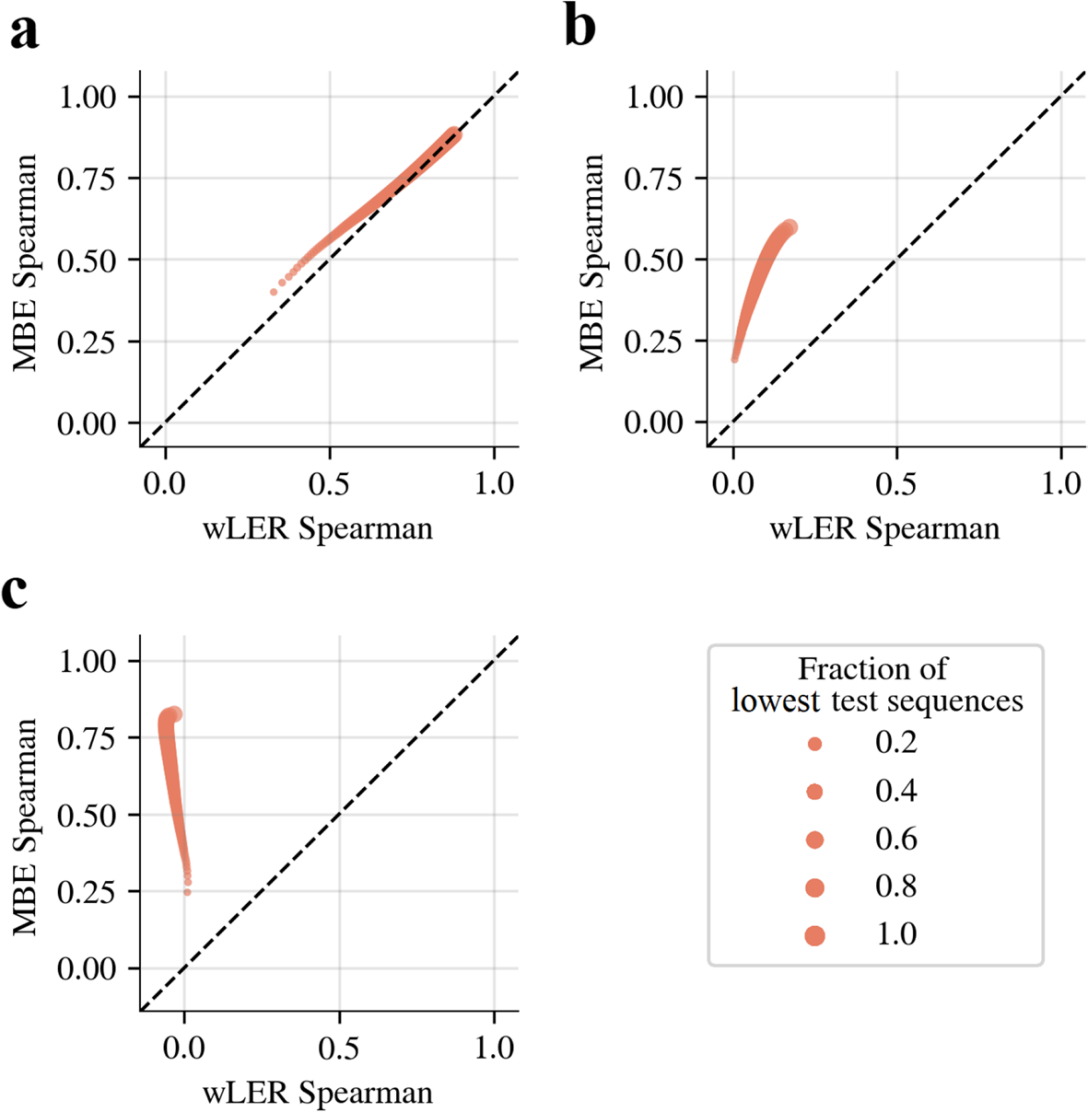
Simulated negative selection prediction results. Comparison of wLER and MBE predictive accuracy for simulated negative selection using the 100-unit NNs on the **a** 21-mer insertion ($4.6 \times 10^7$ short reads), **b** avGFP mutagenesis ($4.6 \times 10^5$ long reads), and **c** AAV recombination ($4.6 \times 10^5$ long reads) datasets. Dot size represents the fraction of test sequences with the lowest ground truth fitness used to compute Spearman correlation. A 0.2-fraction of lowest test sequences corresponds to the sequences in the bottom 20% of all unique test set sequences after ranking by ground truth fitness. To produce each curve, we computed Spearman correlation for every fraction of lowest test sequences between 0.01 and 1.0, inclusive, in 0.01 increments for each method, to obtain a sweeping continuum in the plot, although the legend shows dot sizes only in increments of 0.2 to reduce clutter. The dashed black line represents the equal performance of the two approaches

In negative selection experiments, the property being selected for is opposite from the property of interest. Thus, a key goal is to produce accurate predictions for sequences with low ground truth fitness, for which the post-selection read counts are, by definition, low, making these estimates extremely challenging, particularly for approaches based on cLE estimates—including wLER—that are high variance when sequencing counts are low, and cannot share information across similar but non-identical reads to mitigate this noise. We compared wLER and MBE predictive accuracy using generalized Spearman correlation focused on sequences with low ground truth fitness. MBE achieved higher predictive accuracy, not only overall, but also specifically on the subset of the test sequences with lowest true fitness (Fig. 5).

##### Selection for sequence selectivity

**Fig. 6:**
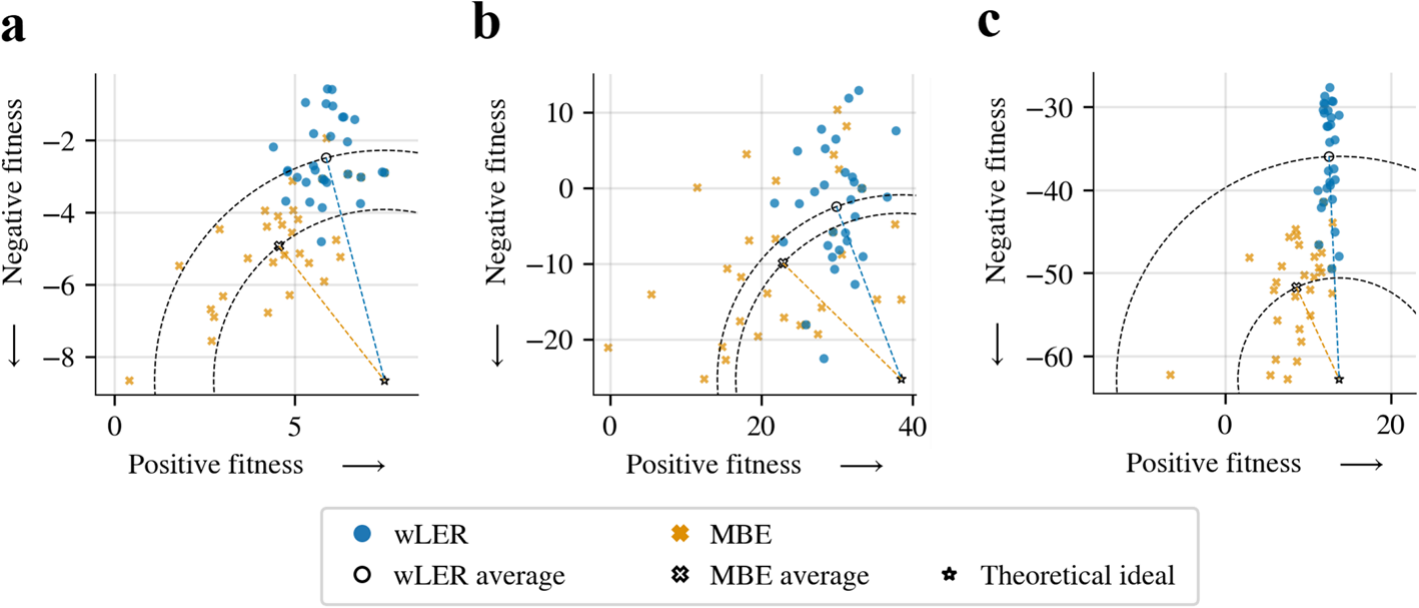
Simulated sequence selectivity prediction results. Comparison of wLER and MBE (using 100-unit NNs) for identifying selective test sequences over three simulated datasets: **a** 21-mer insertion ($4.6 \times 10^7$ short reads), **b** avGFP mutagenesis ($4.6 \times 10^5$ long reads), and **c** AAV recombination ($4.6 \times 10^5$ long reads). Colored points show the true positive and negative fitness of the top ten test sequences identified from each of the three test folds from threefold cross-validation according to each model’s predicted selectivity (i.e., difference in predicted positive and negative fitness values). To gauge overall performance, the average point from each method is also plotted in black-and-white, as is a theoretical optimally selective sequence (star) with the maximum positive fitness and minimum negative fitness among all sequences in the relevant dataset. Distance from optimal to average is conveyed by a circular contour line through the average point for each method; the size of the gap between the two circles is indicative of how much closer MBE is to the optimum than wLER. On all three datasets, MBE is significantly more accurate than wLER at identifying the $1\%$ of test sequences with the highest true selectivity ($p < 10^{-3}$)

A key reason to seek high predictive accuracy for the negative selection task is so that we can leverage this task to perform a selectivity experiment, wherein we seek to identify sequences that simultaneously appear high in a positive fitness and low in the negative fitness. Indeed, we find that MBE is better than wLER at identifying those *selective sequences*, which required a three-condition model for MBE, and a two-output regression for wLER. First, we found that MBE yielded better predictive accuracy than wLER on both the negative fitness (Fig. 5) and the positive fitness (Additional file 1: Fig. S10a, d, and g). More importantly, MBE was also better at identifying the selective sequences, which we assessed as follows. To measure a sequence’s selectivity, we computed the difference between its positive and negative fitness values: the larger this difference, the more selective the sequence is for the positive selection relative to the negative selection. MBE was more accurate than wLER in identifying selective sequences (Fig. 6). Moreover, the best sequences identified by MBE were, on average, closer to a theoretical optimally selective sequence, compared to wLER (Fig. 6, Additional file 1: Fig. S10c, f, and i). Overall, for each of the three datasets, MBE was significantly better than wLER at identifying the $1\%$ of test sequences with the highest true selectivity ($p < 10^{-3}$).

### Overview of experiments with real data

Having characterized the behavior of wLER and MBE in a broad range of simulated settings, we applied these methods on real experimental data. Next, we describe the datasets, model architectures, and evaluation metrics used to empirically compare the methods on experimental data, before presenting the results.

#### Real experimental data

We used five experimental datasets—each comprised of sequencing data from a pre-selection library and after one or more selections on that library. For our evaluations, we also used low-throughput experimental property measurements corresponding to the selected property for each of the five sequencing datasets. Each experimental dataset and its corresponding property measurements are summarized in Table 2 and described briefly in the same order here:

1. A library of 21-mer nucleotide insertions into a fixed AAV background sequence subjected to a round of packaging selection, and packaging titer measurements for five sequences not present in the library [20].
2. A library containing every 15 amino acid peptide in the SARS-CoV-2 proteome (which has 14,439 amino acids) subjected to four rounds of selection for binding to human major histocompatibility complex (MHC). For ground truth, there are $IC_{50}$ measurements for 24 peptides [17].
3. A site saturation mutagenesis library containing all single and double amino acid mutations within the 168 nucleotide IgG-binding domain of protein G (GB1) subjected to selection for binding to IgG-FC. For ground truth, there are $\Delta \text {ln}(K_A)$ measurements for 11 individual variants [12].
4. A library containing natural chorismate mutase homologs and designed sequences sampled from a direct coupling analysis (DCA) model. All sequences are of length 288 nucleotides. For ground truth, there are biochemical measurements for 11 variants [21].
5. A $\beta$-glucosidase enzyme (Bgl3) error-prone PCR random mutagenesis library subjected to a heat challenge and high-throughput droplet-based microfluidic screening. All sequences are of length 1506 nucleotides. For ground truth, there are $T_{50}$ (temperature where half of the protein is inactivated in ten minutes) measurements for six mutants [23].

**Table 2:** Summary of experimental datasets. For each dataset, we list the library description (Library); sequence length in nucleotides (*L*); number of unique library sequences after holding out experimentally validated variants, if needed ($M'$); number of experimentally validated variants (*n*); $\%$ of the sequence of interest covered by individual reads (Cover); number of pre-selection reads ($N^\text {pre}$); and number of post-selection reads ($N^\text {post}$). For the dataset from Huisman et al. [17], the number of reads for each round of selection is presented on a separate row

Library	*L*	*M′*	*n*	Cover	$N^\text {pre}$	$N^\text {post}$
AAV5 insertion [20]	21	8,552,729	5	100	46,049,235	45,306,265
SARS-CoV-2-derived peptide [17]	45	167,841	24	100	44,073	88,032
SARS-CoV-2-derived peptide [17]	45	167,841	24	100	88,032	169,730
SARS-CoV-2-derived peptide [17]	45	167,841	24	100	169,730	235,787
SARS-CoV-2-derived peptide [17]	45	167,841	24	100	235,787	160,863
GB1 double site saturation [12]	168	536,953	11	100	324,434,913	262,112,210
Chorismate mutase homolog [21]	288	3063	11	100	1,228,687	1,929,212
Bgl3 random mutagenesis [23]	1506	468,194	6	100	1,177,842	710,555

Recall that our primary motivation was to improve prediction of LE for unobserved sequences. To assess prediction performance, we held out all sequences in the experimentally measured validation sets from the corresponding sequencing datasets prior to modeling. However, we also assessed LE estimation performance by repeating our experiments without holding out these sequences from the sequencing data, but could only evaluate the estimation task on those sequences that appeared in both the high- and low-throughput experiments for a given protein.

#### Model architectures

For all real experimental datasets (except for Bgl3), we used the smallest NN architecture because it tended to achieve better cross-validation performance than the linear architectures and comparable performance to the larger NN and CNN architectures, while being more parsimonious (Additional file 1: Fig. S11a–l). For the Bgl3 dataset, we used a simpler linear model because overfitting was observed with the NNs (Additional file 1: Fig. S11m–o). For the one dataset that had multiple rounds (the SARS-CoV-2-derived peptide dataset [17]), we viewed the multiple rounds as multiple conditions: we used a multi-output model with one output per round and took the final prediction to be the average of the predictions for each round.

Note that, in practice, when classes are highly imbalanced (i.e., $N^{\text {pre}}$ is much larger than $N^{\text {post}}$, or vice versa), a read-level classifier with an unmodified loss to implement MBE would learn to predict the more prevalent class, which may be undesirable. To counter this effect, we re-weighted each class in the loss function by the total number of reads in each class, to make the impact of the data in each class comparable (Methods). This correction was not necessary in our simulations because the classes there were exactly balanced by construction (i.e., $N^{\text {pre}} = N^{\text {post}}$).

#### Evaluation methods

For the real experimental datasets where ground truth fitness values for the library sequences are unknown, we use available low-throughput (non-sequencing-based) experimental fitness measurements (which may still be corrupted by noise, but are more direct measurements of the property of interest than the sequencing-based assays) for validation. Specifically, we compare the approaches by computing Spearman correlation between predicted LE and low-throughput experimental property measurements. We use a paired *t*-test to assess the statistical significance of the performance difference between wLER and MBE aggregated across all five experimental datasets.

#### Results on real experimental data

**Fig. 7:**
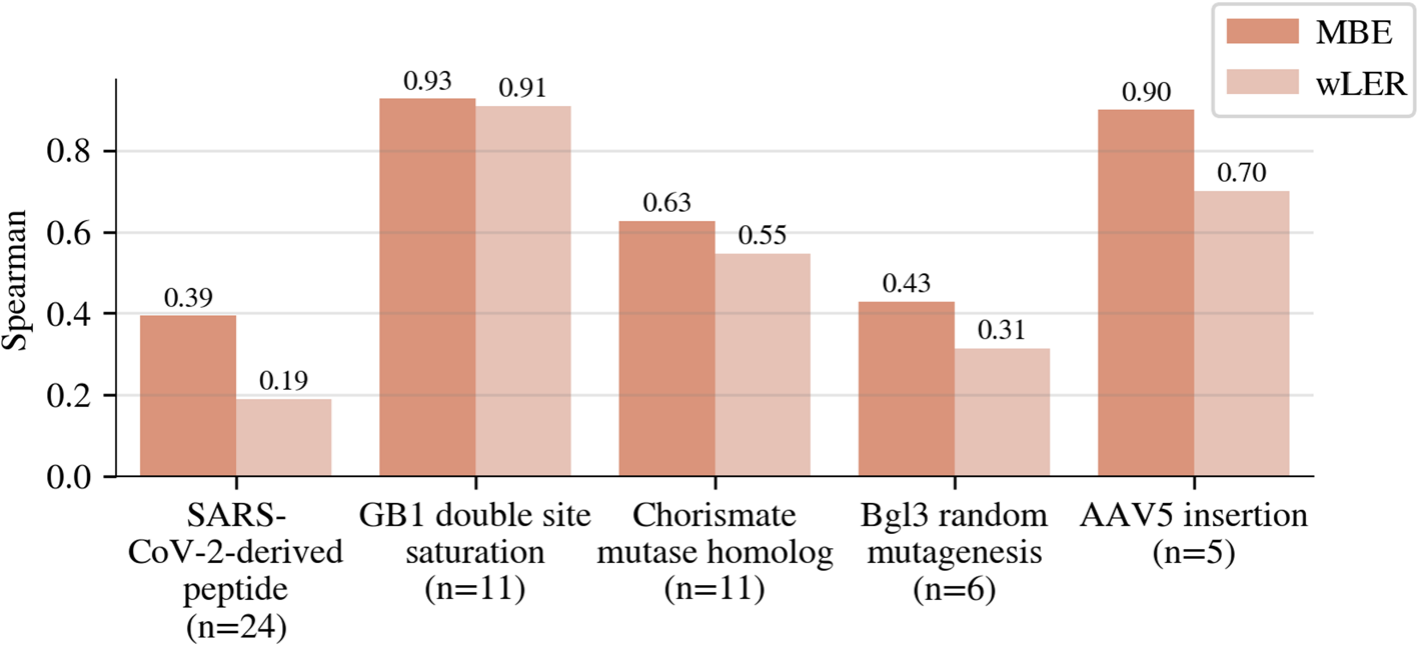
Real experimental prediction results. Comparison of Spearman correlation between wLER or MBE predictions and *n* experimental property measurements from the SARS-CoV-2-derived peptide [17], GB1 double site saturation [12], Chorismate mutase homolog [21], Bgl3 random mutagenesis [23], and AAV5 insertion [20] libraries. Each method is trained on real pre- and post-selection sequencing data, then used to predict the fitness of the *n* unobserved test sequences. The 100-unit NN model architecture is used for all datasets except the Bgl3 dataset, for which the linear architecture with IS features is used. The average performance improvement of MBE over wLER over all five experimental datasets, jointly, is statistically significant ($p < 0.03$). For comparisons of model predictions and property measurements for each experimentally measured sequence for each dataset, see Additional file 1: Fig. S12

Across all the real datasets, MBE achieved better predictive accuracy than wLER (Fig. 7, Additional file 1: Fig. S12) while improving or maintaining estimation accuracy (Additional file 1: Fig. S13). In general, we found that performance differences were smaller for estimation than prediction across all real datasets, but that MBE achieved higher estimation accuracy than wLER whenever there were differences, a promising trend that should be confirmed using larger validation sets in future studies. Moreover, for the SARS-CoV-2 dataset from Huisman et al. [17], predictions of experimental $IC_{50}$ by MBE were more accurate than the predictions by NetMHCIIpan4.0, a model specifically devised to predict peptide binding to MHC II molecules (Additional file 1: Table S1). An important challenge when working with experimental data is that, to obtain the best ground truth values possible, we require access to detailed biophysical assays rather than sequencing-based proxies. Consequently, the validation data we have access to have extremely limited sample sizes (ranging from 5 to 24 test points), thereby limiting our ability to detect statistical significance on each dataset individually. Nevertheless, the trends that we observed on the simulated data continue on each dataset, and when predictive performance over all of them is considered jointly, the improvement of MBE over wLER is statistically significant ($p < 0.03$) (Fig. 7).

## Discussion

Quantitatively characterizing the difference in sequence abundances between two conditions using high-throughput sequencing data—as occurs, for example, in high-throughput selection experiments—is a key component in answering a large range of scientific questions. Not only do we wish to quantify the differences in observed data, but we also often want to predict the difference for sequences not yet observed—for example, in order to design further rounds of experimentation. Until now, quantification was accomplished by counting the number of times a sequence occurred in each condition and taking the ratio of these counts (after normalization). Then, optionally, one may have constructed a regression model to predict these count-based log-enrichment ratios. A key limitation permeating all of this processing is the inability of count-based estimates to share any information across sequences that are not identical, even though such sharing of information can be extremely valuable. Herein, we introduce and evaluate a framework that overcomes this key limitation. Our framework is based on a reformulation of the problem that uses density ratio estimation, implemented using any standard machine learning classifier. Our new method, model-based enrichment, improves performance over competing approaches across a broad range of simulated data, as well as on real experimental data. As more experimental data become available, it will be valuable to perform further characterization of MBE.

MBE enables estimation of log-enrichment in challenging experimental setups comprised of, for example, short reads spanning a sequence of interest; long reads with poor coverage; a mixture of both short and long reads; and settings with more than two conditions—such as when we seek to find selective sequences enriched for one selection and negatively selected by another, as occurs in engineering gene therapy viral vectors to selectively infect one cell type but not another. In general, our approach also helps to mitigate poor estimates arising from low statistical power emerging from, say, access to only a small amount of sequencing data.

Our newly developed method, MBE, can immediately leverage any advances in general machine learning classifiers, and naturally handles sequencing reads of variable lengths within a given experiment, so long as the classifier itself does so—as we demonstrated herein using convolutional neural networks. In principle, the predictive performance of such variable-length classifiers can potentially be further improved by incorporating other informative inputs—in addition to read sequence. For example, in settings where it is possible to align to a known reference sequence before modeling, one may supply the mapped position for each read as an additional input. Moreover, it is straightforward to incorporate additional machine learning advances for probabilistic classifiers into model-based enrichment, such as calibration methods that ensure predicted class probabilities are interpretable as a metric of confidence (Additional file 1: Supplementary Note 2).

## Conclusions

Differential sequencing analysis is a prevalent and important component in addressing scientific questions across a large range of biological application areas, where the goal is often not only to quantify differences in sequence abundances for the observed data, but also for sequences not yet observed. Until now, quantification was accomplished by computing count-based log-enrichment—by counting the number of times a sequence occurred in each condition and taking the ratio of these counts—and, optionally, constructing a regression model to predict these count-based log-enrichment ratios. Herein, we introduce and evaluate a new framework, model-based enrichment, that overcomes a key limitation of count-based approaches. This framework is based on sound theoretical principles and can be implemented using any standard machine learning classifier. Our new method improves performance over competing approaches based on either raw counts or weighted regression on count-based log-enrichment, and enables estimation of log-enrichment in challenging experimental setups. In particular, we show this improvement holds across a broad range of simulated data, as well as on real experimental data, and that our approach helps to mitigate poor estimates arising from relatively little sequencing data. We anticipate that, as high-throughput selection experiments and sequencing-based assays continue to become more varied in their applications, the full potential of MBE will be further revealed.

## Methods

### Log-enrichment regression

Subjecting two sequence libraries—one for each of two conditions *A* and *B*—to high-throughput sequencing yields a dataset

1 $$\begin{aligned} \mathcal {D} = \{ (r_i, y_i) \}_{i=1}^{M} \end{aligned}$$

where $r_i$ is the $i^{\text {th}}$ read’s sequence and $y_i$ is a binary $-1/+1$ label indicating whether read $r_i$ arose from condition *A* or *B*, respectively. In our analyses of high-throughput selection experiments, the conditions *A* and *B* correspond to pre- and post-selection, but the following methodology applies broadly to settings with sequencing data from two conditions for which we seek to understand or predict sequence properties. In subsequent sections, we also further generalize to more than two conditions.

From these data, $\mathcal {D}$, one often calculates a count-based log-enrichment (cLE) estimate for each unique sequence [2, 3, 5, 10, 11], which serves as an estimate of the extent to which the sequence has the property being investigated. In selection experiments, we refer to the selection process as acting according to a particular *fitness*, and the cLE estimate thus serves as a proxy for this fitness. To compute cLE estimates, it is convenient to represent $\mathcal {D}$ in terms of unique sequences: $\mathcal {D}' = \{ (x_i, n_i^A, n_i^B) \}_{i=1}^{M'}$ where $\{ x_i \}_{i=1}^{M'} \subseteq \{ r_i \}_{i=1}^M$ is the set of unique observed sequences,

2 $$\begin{aligned} n_i^A = \sum _{(r, y) \in \mathcal {D}} \mathbbm {1}{\{r = x_i\}} \mathbbm {1}{\{y = -1\}} \end{aligned}$$

is the observed read count for sequence $x_i$ in the sequencing data for condition *A*, and

3 $$\begin{aligned} n_i^B = \sum _{(r, y) \in \mathcal {D}} \mathbbm {1}{{\{r = x_i\}} \mathbbm {1}{\{y = +1\}}} \end{aligned}$$

is the corresponding condition *B* read count. For each sequence, the cLE estimate is equal to the log-ratio of read frequencies for conditions *A* and *B*:

4 $$\begin{aligned} \log e_i = \log \left( \left( \frac{n_i^B}{N^B}\right) \left( \frac{n_i^A}{N^A}\right) ^{-1} \right) , \end{aligned}$$

where $N^A = \sum _{i=1}^{M'} n_i^A$ and $N^B = \sum _{i=1}^{M'} n_i^B$. In practice, it is common to add a small constant to each count prior to calculating cLE estimates for mathematical convenience [2, 5]. These “pseudo-counts” stabilize the cLE estimates, and allow one to avoid division by zero for sequences observed in only one condition. In our experiments, we added a pseudo-count of 1 to each raw count.

Log-enrichment (LE) regression approaches fit a model that maps from $x_i$ to $\log e_i$. In particular, Zhu et al. [20] derive a weighted least squares procedure for fitting such a regression model; their procedure assigns a weight, $w_i = (2 \sigma _i^2)^{-1}$, to each sequence, where

5 $$\begin{aligned} \sigma _i^2 = \frac{1}{n_i^B} \left( 1 - \frac{n_i^B}{N^B}\right) + \frac{1}{n_i^A} \left( 1 - \frac{n_i^A}{N^A}\right) . \end{aligned}$$

This choice of $w_i$ is motivated by a convergence argument: $\sigma _i^2$ is the asymptotic variance of $\log e_i$ [11, 20]. Note that when the counts $n_i^A$ and $n_i^B$ are low, $\log e_i$ is a noisier estimate of fitness and the corresponding weight, $w_i$, is smaller. Thus, training a model $f_\theta$, with learnable parameters $\theta$, using the weighted least squares loss

6 $$\begin{aligned} \ell _{\text {R}} = \sum _{i=1}^{M'} w_i (\log e_i - f_\theta (x_i))^2 \end{aligned}$$

accounts for the heteroscedastic noise in the observed cLE estimates. We refer to this modeling approach as the weighted LE regression (wLER) approach.

### Model-based enrichment

Existing methods that use cLE estimates to train predictive models [20, 22, 29, 32] proceed in two steps: first, one computes a cLE estimate for each observed sequence, and, second, one uses supervised regression to train a model to predict these cLE estimates given the observed sequences. Here, we present a new method, model-based enrichment (MBE) that performs both of these steps at once by reframing LE estimation as a density ratio estimation (DRE) problem. First, we define the density ratio between libraries in each of two conditions and show that a cLE estimate can be viewed as an approximation of the density ratio. Then, we describe the technical details of the MBE approach, which uses a probabilistic classifier trained on sequencing reads to perform DRE.

As in the preceding section, suppose two libraries corresponding to conditions *A* and *B* have been subjected to high-throughput sequencing. Each library can be represented by a discrete probability distribution over sequences: each unique sequence $x_i$ is present in the libraries from conditions *A* and *B* in some ground truth proportions $p^A(x_i), p^B(x_i) \in [0, 1]$. The density ratio between these two library distributions is $d = \frac{p^B}{p^A}$.

We can connect this density ratio to cLE estimates. The cLE estimate, $\log e_i$, is the log-ratio of the two empirical read frequencies $\frac{n_i^A}{N^A}$ and $\frac{n_i^B}{N^B}$ (Eq. 4). These read frequencies are approximations of the true library proportions $p^A(x_i)$ and $p^B(x_i)$ based on the observed sample of sequencing reads. Thus, the cLE estimate, $\log e_i$, can be viewed as a sample-based approximation of $\log d(x_i)$. LE regression methods can, therefore, be viewed as, first, approximating $\log d$ using observed counts, and then training a regression model to predict these approximate log-density ratios.

In contrast, DRE techniques [40, 41] can be used to model the density ratio directly from sequencing data. Our proposed MBE approach uses a classification-based DRE technique [38–41] which involves training a probabilistic classifier, $g_\theta$, on $\mathcal {D}$ (Eq. 1) to predict $y_i$ from $r_i$ for each individual read. We use the standard logistic loss,

7 $$\begin{aligned} \ell _{\text {C}} = \sum _{i=1}^M \log (1 + \exp (-y_i g_\theta (r_i))). \end{aligned}$$

By minimizing this loss with respect to $\theta$ to obtain the maximum likelihood estimate, $\hat{\theta }$, we obtain our predictive model, $g_{\hat{\theta }}(x)=p(y \mid r=x)$. By Bayes’ theorem, we can also derive an estimator of the density ratio [39, 41] as follows:

8 $$\begin{aligned} d(x)&= \frac{p^B(x)}{p^A(x)} = \left( \frac{p(y=+1 \mid r=x) p(x)}{p(y=+1)}\right) \left( \frac{p(y=-1 \mid r=x) p(x)}{p(y=-1)}\right) ^{-1}\end{aligned}$$

9 $$\begin{aligned}&= \frac{p(y=+1 \mid r=x)}{p(y=-1 \mid r=x)} \frac{p(y=-1)}{ p(y=+1)} \approx \frac{N^A g_{\hat{\theta }}(x)}{N^B (1 - g_{\hat{\theta }}(x))}, \end{aligned}$$

where $N^A$ and $N^B$ are the total read counts for each condition, as in Eq. 4. This ratio of predicted class probabilities, $\frac{N^A g_{\hat{\theta }}}{N^B (1 - g_{\hat{\theta }})}$, provably converges to *d* [39–41] and is the optimal density ratio estimator among a broad class of semi-parametric estimators (that includes the wLER method) in terms of asymptotic variance under a correctly specified model [39] (Additional file 1: Supplementary Note 1).

MBE naturally accounts for heteroscedastic noise in the observed sequencing data. To see this, we can rewrite $\ell _{\text {C}}$ in terms of unique sequences,

10 $$\begin{aligned} \ell _{\text {C}} = \sum _{i=1}^{M'} n_i^B \log (1 + \exp (-g_\theta (x_i))) + n_i^A \log (1 + \exp (g_\theta (x_i)), \end{aligned}$$

where $n_i^A$ and $n_i^B$ are read counts as defined in Eq. 2 and 3. This form of $\ell _{\text {C}}$ highlights the fact that sequences with higher counts make larger contributions to the loss than those with lower counts, simply by virtue of having been sequenced many times. Thus, $g_\theta$ is biased towards modeling *d* more accurately for sequences with more sequencing data, as desired. In this way, the MBE approach accounts for heteroscedasticity in the observed sequencing data without the need to derive a bespoke weighted loss function, unlike the wLER approach.

Note that, in practice, when classes are highly imbalanced (i.e., $N^A$ is much larger than $N^B$, or vice versa), a read-level classifier with an unmodified logistic loss will learn to predict the more prevalent class, which here may be undesirable. To counter this effect, one can use standard machine learning techniques for training classification models under class imbalance, such as class weighting, wherein samples from the minority class are up-weighted in the loss—typically the negative log-likelihood—so that each class has equal overall contribution to the loss. Such techniques were not required in our simulations because classes were always exactly balanced by construction (i.e., $N^A = N^B$). In our experiments on real sequencing data, however, we re-weighted each class in the logistic loss (Eq. 10) by the total number of reads in each class to make the impact of the data in each class comparable, as follows:

11 $$\begin{aligned} \ell _{\text {C}} = \text {max} \{ N_A, N_B \} \sum _{i=1}^{M'} \frac{n_i^B }{N_B} \log (1 + \exp (-g_\theta (x_i))) + \frac{n_i^A }{N_A} \log (1 + \exp (g_\theta (x_i)). \end{aligned}$$

### Multi-output modeling

In practice, one often aims to compare sequences across more than two conditions. For example, one may wish to perform multiple rounds of selection for a property of interest (e.g., [17]) or to select for multiple different properties (e.g., [8]). Here, we describe generalizations of the MBE and wLER approaches that can be used to model high-throughput sequencing data collected from more than two conditions. In this setting, one has sequencing data $\mathcal {D}'' = \{ (r_i, y_i) \}_{i=1}^{M}$ where $r_i$ is the $i^{\text {th}}$ read’s sequence and $y_i$ is a categorical label indicating the condition from which the read $r_i$ arose. For example, if one runs an experiment selecting for $k \in \textbf{N}$ different properties, one can define $y \in \{0, 1, \ldots , k\}$ where $y_i = 0$ indicates a read from the pre-selection sequencing data, and $y_i = j$ for $j \in \{1, \ldots , k\}$ indicates a read from the post-selection sequencing data for the $j^{\text {th}}$ property.

It is straightforward to handle multiple conditions using the MBE approach: instead of using a binary classifier, one trains a multi-class classification model, $g_\theta$, to predict the categorical label $y_i$ from read sequence $r_i$ using a standard categorical cross-entropy loss. This produces a model of $p(y \mid r)$ which can be used to estimate the density ratios $d^j = \frac{p^j}{p^0} \approx \frac{N^0 g_\theta ^j}{N^j g_\theta ^0}$, where $p^j$ denotes the true probability distribution corresponding to the library in the $j^\text {th}$ condition and $g_\theta ^j$ denotes the predicted class probability for $y=j$.

For the wLER approach, the data $\mathcal {D}''$ can be converted into cLE estimates for each unique sequence:

$$\begin{aligned} \log e_i^j = \log \left( \left( \frac{n_i^j}{N^j}\right) \left( \frac{n_i^0}{N^0}\right) ^{-1} \right) , \end{aligned}$$

where $n_i^j$ is the number of times the sequence $x_i$ appeared in the sequencing data for the $j^{\text {th}}$ condition and $N^j$ is the total number of reads from the $j^{\text {th}}$ condition. One can, then, fit a multi-output regression model that jointly predicts the cLE estimates for each condition from sequence. The overall loss for training a such a multi-output model, $f_\theta$, using wLER is

$$\begin{aligned} \sum _{j=1}^k \sum _{i=1}^{M'} w_i^j (\log e_i^j - f_\theta ^j(x_i))^2 \end{aligned}$$

where $w_i^j$ is the weight for the $i^\text {th}$ sequence and $j^\text {th}$ condition, and $f_\theta ^j$ denotes the $j^\text {th}$ model output.

### Model architectures and training

In our experiments, we aim to compare and contrast the general performance of the wLER and MBE approaches across a broad range of settings. To enable direct comparison of the two methods, we implemented wLER and MBE using the same model architectures and hyperparameters for the underlying regression and classification models. We will, next, describe each model architecture and provide implementation details.

We used eleven different model architectures: seven architectures that are the same as in Zhu et al. [20]—three linear models and four fully-connected neural networks (NNs)—and four convolutional neural network (CNN) models that can operate on variable-length inputs. The linear models each use one of three input representations: (1) an “independent site” (IS) representation comprised of one-hot encodings of individual amino acids, (2) a “neighbor” representation comprised of the Independent Sites features and one-hot encodings of the pairwise interactions between pairs of positions directly adjacent in sequence, and (3) a “pairwise” representation comprised of the IS features and one-hot encodings of all possible pairwise interactions. All NNs use IS input features and have two hidden layers, and differ by the number of hidden units: 100, 200, 500, or 1000 units per layer. The CNNs differ in the number of convolutional layers used (2, 4, 8, or 16), but all use IS input features, convolutions with a window of size 5 and 100 filters, residual and skip connections, and a global max pooling layer as the penultimate layer.

All models were trained using the AMSGrad Adam optimizer [51] with default learning rate ($10^{-3}$) for ten epochs. For the linear models and NNs, we used the default value for Adam’s $\epsilon$ parameter ($10^{-7}$); for the CNNs, we set $\epsilon =10^{-4}$ and applied gradient clipping with a threshold of 1. We performed threefold cross-validation at the sequence level: for each fold, one third of the unique sequences in the library (and their corresponding sequencing reads) were held-out as a test set.

### Simulating ground truth fitness

We constructed several simulated datasets to help analyze the strengths and weaknesses of MBE, wLER, and cLE across different practical settings. These simulations were motivated by high-throughput selection experiments [8, 20, 31] which perform a selection on large sequence libraries for a property of interest, such as fluorescence [31]. To simulate such selection experiments, we first simulate the ground truth *fitness function* that maps sequence to property, then use this fitness to simulate selection. In the remainder of this section, we describe the process used to simulated fitness as a linear function of independent amino acid sites and randomly selected higher-order epistatic interactions. In the following section, we describe the procedure to simulate selection using simulated fitness.

First, we give a brief overview of the process used to simulate ground truth fitness before providing the technical details. For a given sequence of interest, we first constructed a set containing all independent amino acid sites and a user-specified number of combinations of sites—such as an epistatic combination of the second, third, and tenth positions—drawn randomly from among all possible higher-order epistatic interactions between positions. The degree of each epistatic effect (2 up to the sequence length) is drawn randomly based on an empirical estimate of this degree distribution. The fitness function is, then, taken to be a linear function of all the independent sites and epistatic terms in this constructed set with random coefficients.

In more detail, for a sequence *x* of length $L_a$ amino acids, we simulated the fitness function, $F_T(x)$ as

12 $$\begin{aligned} F_T(x) = \sum _{J \in \mathcal {E}_T} \beta _J \cdot \phi (x[J]), \end{aligned}$$

where *T* is the hyper-parameter controlling the maximum number of epistatic terms included in $F_T$; $\mathcal {E}_T \subseteq 2^{\{ 1, \ldots , L_a\} }$, is a set of index sets—each of which represents an independent site or a particular higher-order epistatic combination—whose construction is described below; *x*[*J*] is the subsequence of *x* at the positions in the index set *J*; $\phi$ denotes standard one-hot encoding; and the coefficients are sampled according to $\beta _J \sim \mathcal {N}(\textbf{0}, 2^{-\mid J \mid } \textbf{I})$.

We constructed $\mathcal {E}_T$ (the specific set of first-order and higher-order epistatic terms to include in the simulated fitness function) to contain all singleton sets ($\{ \{i\} \; \mid \; i \in \{1, \ldots , L_a \} \} \subseteq \mathcal {E}_T$), so that $F_T$ includes terms for all independent sites. In addition, $\mathcal {E}_T$ contains *T* randomly chosen non-singleton index sets, each generated by:

1. Randomly choosing the order of epistasis, *R*, by sampling $\tilde{R} \sim \text {N}(3, 1/2)$ (based on visual inspection of the empirical bell-shaped distribution of the orders of statistically significant epistatic terms in Poelwijk et al.. [29]), and taking $R = \text {round}(\tilde{R})$; and
2. Choosing the specific positions included in the epistatic term by sampling *R* times without replacement from $\{1, \ldots , L_a\}$.

To guide our choice of *T*, we combined the following insights: (i) for a fluorescent protein with 13 amino acids, 260 epistatic terms are sufficient for an accurate model of fitness [29]; (ii) the number of contacts in a protein scales linearly with sequence length [45, 46]; and (iii) recent work suggests that the sparsity of higher-order epistatic interactions in fitness landscapes is closely related to structural contact information [47]. We, therefore, hypothesized that the linear scaling $T = \frac{260 L_a}{13}$ provides a reasonable starting point for analyses.

### Simulating pre- and post-selection sequencing data

The wLER and MBE approaches both aim to accurately quantify sequences of interest based on high-throughput sequencing data. We used simulated high-throughput selection datasets to compare each method’s ability to quantify sequences accurately using sequencing data, which requires simulating sequencing reads from pre- and post-selection libraries. Here, we detail the process of simulating sequencing reads given library sequences and a ground truth fitness function. Then, in the subsequent sections, we will describe how we combined this process with three specific approaches for simulating library sequences to construct our datasets.

Let $\{(x_i, c_i)\}_{i=1}^{M'}$ be pairs of, respectively, a unique library sequence and its true count—as generated, for example, by one of the three library construction simulations described in the subsequent sections. In addition, let $F_T$ be a ground truth fitness function simulated as in the previous section. Briefly, the process to simulate sequencing reads from pre- and post-selection libraries proceeds as follows: first, we generate a pre-selection library distribution by adding a small random perturbation to the empirical distribution $\left\{ c_i / \sum _{i=1}^{M'} c_i \right\} _{i=1}^{M'}$. This step simulates slight distributional perturbations that may occur with PCR amplification, and also has the nice side-effect of allowing one to generate multiple replicates with slightly different pre- and post-selection library distributions for the same set of unique sequences $\{x_i\}_{i=1}^{M'}$. Next, we simulate selection according to the fitness $F_T$: the post-selection library distribution is determined by scaling the pre-selection distribution using $\{\exp (F_T(x_i))\}_{i=1}^{M'}$, which ensures that the ground truth log-density ratio is proportional to the specified fitness $\left( \log d = \log \frac{p^\text {post}}{p^\text {pre}} \propto F_T \right)$. Finally, we sample from the pre- and post-selection distributions to simulate sequencing reads, optionally truncating each read to 100 amino acids uniformly at random to generate short reads.

In more detail, we simulated pre- and post-selection sequencing data by:

1. Sampling $$(p^\text {pre}(x_i))_{i=1}^{M'} \sim \text {Dirichlet}(c_1, \ldots , c_{M'})$$;
2. Setting
  $$\begin{aligned} p^\text {post}(x_i) = Z \exp (F_T(x_i)) p^\text {pre}(x_i) \end{aligned}$$
  where $Z = \sum _{i=1}^{M'} \exp (F_T(x_i)) p^\text {pre}(x_i)$ is a normalization constant;
3. Sampling pre- and post-selection sequencing counts according to
  $$\begin{aligned}&(n_i^\text {pre})_{i=1}^{M'} \sim \text {Multinomial}(N^\text {pre}, (p^\text {pre}(x_i))_{i=1}^{M'}) \quad \text { and } \\&(n_i^\text {post})_{i=1}^{M'} \sim \text {Multinomial}(N^\text {post}, (p^\text {post}(x_i))_{i=1}^{M'}) \end{aligned}$$
  for some desired number of sequencing reads, $N^\text {pre}$ and $N^\text {post}$; and, if simulating short reads, additionally
4. Sampling $n_i^\text {pre}$ and $n_i^\text {post}$ contiguous 100-mers from $x_i$ uniformly at random.

### Simulated insertion libraries

To empirically compare and contrast our MBE approach to the wLER approach in practical settings, we sought to simulate realistic sequence libraries motivated by experimental constructions from recent studies.

We simulated diversified libraries of insertion sequences motivated by our work in adeno-associated virus (AAV) capsid engineering [20]. In this study, we used a library of 21-mer nucleotide insertion sequences, where each codon was independently sampled from the distribution defined by the NNK degenerate codon: “NN” denotes a uniform distribution over all four nucleotides in the first two positions of a codon and “K” denotes equal probability on nucleotides G and T in the third codon position. Here, we sampled sequences from this NNK distribution to simulate three insertion libraries containing length 21, 150, and 300 nucleotide sequences, respectively. Specifically, each sequence is generated by sampling either 7, 50, or 100 codons independently from the NNK distribution. To keep each of our simulated insertion datasets as similar as possible to the experimental data from Zhu et al. [20], we sampled sequences in this manner until we obtained a set of $8.5 \times 10^6$ unique library sequences. We take the set $\{(x_i, c_i)\}_{i=1}^{8.5 \times 10^6}$ to be the simulated library, where $x_i$ is the $i^{\text {th}}$ unique insertion sequence and $c_i$ is the number of times it was sampled from the NNK distribution before $8.5 \times 10^6$ unique sequences were generated.We used *T* = 140, 1000, and 2000 to simulate ground truth epistatic fitness for the 21-mer, 150-mer, and 300-mer insertion libraries, respectively, and simulated $N^\text {pre} = N^\text {post} = 4.6 \times 10^7$ sequencing reads for each library using the process described in the previous section.

To gain insight into the effect of sequencing error on MBE and wLER, we also constructed a noisy version of the sequencing data for the 21-mer insertion library containing simulated sequencing errors in both the pre- and post-selection sequencing reads. Because Illumina’s next-generation sequencers have an approximately 0.1% error rate and predominantly produce substitution errors [49], we added substitution errors to each position of each simulated read uniformly at random with probability 0.001.

### Simulated avGFP mutagenesis library

Motivated by a recent study of the fitness landscape of the green fluorescent protein from *Aequorea victoria* [31], we generated an avGFP library by mutating positions of the avGFP reference sequence from Sarkisyan et al. [31] (238 amino acids long) uniformly at random. We used a mutation rate of 10% to generate $2.5 \times 10^7$ unique library sequences. Specifically, we generated mutated avGFP sequences—by mutating each position independently with probability 0.01—until we obtained a set $\{(x_i, c_i)\}_{i=1}^{2.5 \times 10^7}$, where each $x_i$ a unique library sequence and $c_i$ is the number of times it was generated before $2.5 \times 10^7$ unique sequences were obtained.

To simulate selection and sequencing, we used $T=4,760$ to simulate ground truth fitness, and generated both long-read ($N^\text {pre} = N^\text {post} = 4.6 \times 10^5$ to be within PacBio’s throughput [34, 35]) and short-read ($N^\text {pre} = N^\text {post} = 4.6 \times 10^7$ to match the dataset from Zhu et al. [20]) sequencing data.

### Simulated AAV recombination library

We simulated a recombination library of AAV capsid sequences motivated by an AAV-directed evolution study [8], wherein several AAV serotypes are recombined using seven crossovers separating eight recombination blocks. We generated library sequences by recombining AAV serotypes 1-9 with seven uniformly spaced crossovers. This library contains 26,873,856 unique library sequences that are 2253 nucleotides long. We simulated epistatic fitness with $T=15,020$.

To assess the effects of the type and amount of sequencing data, we generated multiple datasets: three long-read datasets with $N^\text {pre} = N^\text {post} = 4.6 \times 10^3$, $4.6 \times 10^4$, and $4.6 \times 10^5$, respectively; one short-read dataset with $N^\text {pre} = N^\text {post} = 4.6 \times 10^7$; and one hybrid dataset containing $4.6 \times 10^3$ long reads and $4.5 \times 10^7$ short reads for both pre- and post-selection. To help gain insights into the effects of sequencing error, we also constructed a noisy AAV recombination dataset that incorporated simulated sequencing errors into $4.6 \times 10^5$ pre- and post-selection sequencing reads using SimLoRD [50] to simulate PacBio SMRT sequencing errors.

### Supplementary information

**Additional file 1.** Supplementary Notes and Figures contains theoretical results relevant to classifier-based density ratio estimation and results of additional experiments evaluating model-based enrichment’s empirical performance.

**Additional file 2.** Review history.

## Data Availability

A general Python implementation of model-based enrichment and source code for simulated datasets, training models, and evaluating performance are available under the MIT open source license at https://github.com/apbusia/model_based_enrichment [52] and https://github.com/apbusia/selection_dre [53] (DOI: 10.5281/zenodo.8298055 [54]). The experimental selection datasets from Zhu et al. [20], Huisman et al. [17], Olson et al. [12], and Russ et al. [21] are available as supplementary files and/or upon request. The experimental dataset from Romero et al. [23] is also available on Github [55].
