# Supplementary material for: MBE: model-based enrichment estimation and prediction for differential sequencing data
Source: Genome Biol. 2023 Oct 2;24:218. doi: 10.1186/s13059-023-03058-w (PMC10544408; doi:10.1186/s13059-023-03058-w)
Supplement: Supplementary file 1 — Additional file 1. Supplementary Notes and Figures contains theoretical results relevant to classifier-based density ratio estimation and results of additional experiments evaluating model-based enrichment’s empirical performance. [file 13059_2023_3058_MOESM1_ESM.pdf]

Additional File 1: Supplementary Notes and Figures

MBE: Model-based enrichment estimation and prediction for differential sequencing data

Supplementary Note 1: Asymptotic optimality of model-based enrichment

In this section, we review key parametric convergence results which imply that, under the assumption of a correctly specified parametric model, the proposed model-based enrichment (MBE) estimator is optimal among a broad class of semi-parametric density ratio estimators—including the weighted log-enrichment regression (wLER) method [1]—in terms of asymptotic variance.

We begin by recalling some notation: let p^A and p^B be two probability distributions, $d = \frac{p^B}{p^A}$ be their density ratio, and

$$\mathcal{D} = \{(r_i, y_i)\}_{i=1}^{M} \tag{1}$$

be a dataset of observed samples where y_i is a binary label indicating whether the sample r_i is from p^A ($y_i = -1$) or p^B ($y_i = +1$). Further, let N^A and N^B be the number of samples from p^A and p^B, respectively. Recall that the MBE approach uses logistic regression to learn a classifier that predicts $p(y_i \mid r_i)$, and these predicted class probabilities give an estimate of the density ratio (Methods). In other words, the MBE approach estimates the density ratio using the parametric model

$$\log d_\theta(r) = \theta_0 + \phi_{\theta_1}(r) \tag{2}$$

where $\theta_0 \in \mathbf{R}$, $\theta = (\theta_0, \theta_1) \in \mathbf{R}^b$ is a b-dimensional parameter, and ϕ_{θ_1} is a real-valued function (*e. g.,* defined by the choice of model architecture).

Whenever correctly-specified density models for both p^A and p^B are unavailable, direct density ratio estimation of $\frac{p^B}{p^A}$—as performed by the MBE approach—is preferable compared to separate density estimation of p^A and p^B in terms of asymptotic unnormalized Kullback–Leibler divergence to the true density ratio, d [2]. Moreover, Qin [3] showed that, if the

logistic regression model is correctly specified—that is, if the true density ratio d is realized by d_{θ^*} in the parametric model—then the MBE approach is optimal among a large class of semi-parametric density ratio estimators in the sense that it has the smallest asymptotic variance. Specifically, the class of semi-parametric estimators in Qin's analysis is a class of generalized moment-matching estimators:

$$\{\hat{\theta}_\eta \mid \eta_\theta(r) \in \mathbb{R}^b, \mathrm{Var}_{p^A}[\eta_\theta(r)] \text{ and } \mathrm{Var}_{p^B}[\eta_\theta(r)] \text{ are finite,}$$
$$\frac{1}{N^A} \sum_{(r_i, y_i) \in \mathcal{D}} \eta_{\hat{\theta}_\eta}(r_i) d_{\hat{\theta}_\eta}(r_i) \mathbb{1}\{y_i = -1\} = \frac{1}{N^B} \sum_{(r_i, y_i) \in \mathcal{D}} \eta_{\hat{\theta}_\eta}(r_i) \mathbb{1}\{y_i = +1\}\}.$$

This class of estimators contains several popular density ratio estimators, including the Kullback-Leibler (KL) importance estimation procedure [2, 4] that learns a density ratio model by minimizing empirical KL divergence between $d \cdot p^A$ and p^B. Other estimation techniques, including weighted and non-linear least squares regression, can also be cast in terms of generalized moment-matching optimization [5] and, therefore, the wLER approach is included in Qin's class of estimators, as are several other existing log-enrichment regression approaches [6, 7]. Thus, under a correctly specified parametric model, the MBE approach is the preferred density ratio estimation technique—and, in the context of this work, the preferred technique for quantifying sequences based on sequencing data from a high-throughput screen or selection—in terms of asymptotic variance.

Supplementary Note 2: Statistical guarantees for model-based enrichment

Model-based enrichment (MBE) leverages probabilistic classification to predict log-enrichment (LE) values for individual sequences of interest. Although in many applications—such as the design of large sequence libraries as in Zhu *et al.* [1]—it may be sufficient to produce LE values with the correct rank order, there may be settings where it is desirable to also have a notion of confidence for individual LE predictions. For machine learning probabilistic classifiers, the predicted class probabilities for a given input provide a human-interpretable confidence. When the classifier's probabilities are *calibrated*, this confidence can be interpreted with particular statistical guarantees [8–10]. For example, if a calibrated classifier produces a prediction of 0.8 for class A, then the calibration yields the statistical guarantee that over all instances classified at 0.8 by the classifier, that precisely 80% of them are truly class A. Detailed discussion and implementation of specific calibration methods [9] is beyond the scope of the current work, but existing methods can be directly applied to the classifier in MBE—including those for modern-day neural network and convolutional model architectures [10]—so that the underlying classification model is well-calibrated. As such, MBE can easily be applied so that it produces probabilities

that can be interpreted as a valid metric of confidence, in addition to an accurate rank-ordering of sequences.

Supplementary figures and tables

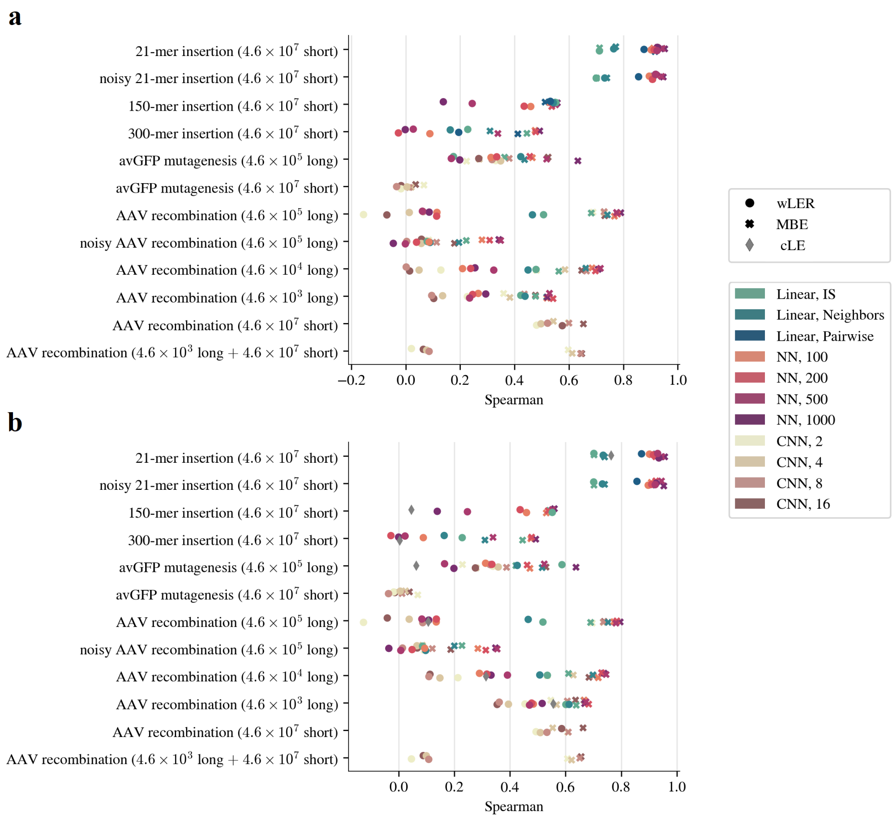

Fig. S1 Simulation results for all model architectures. (a) and (b) are the same as Fig. 4a and b, respectively, but display the Spearman correlation between model predictions and ground truth fitness for all model architectures.

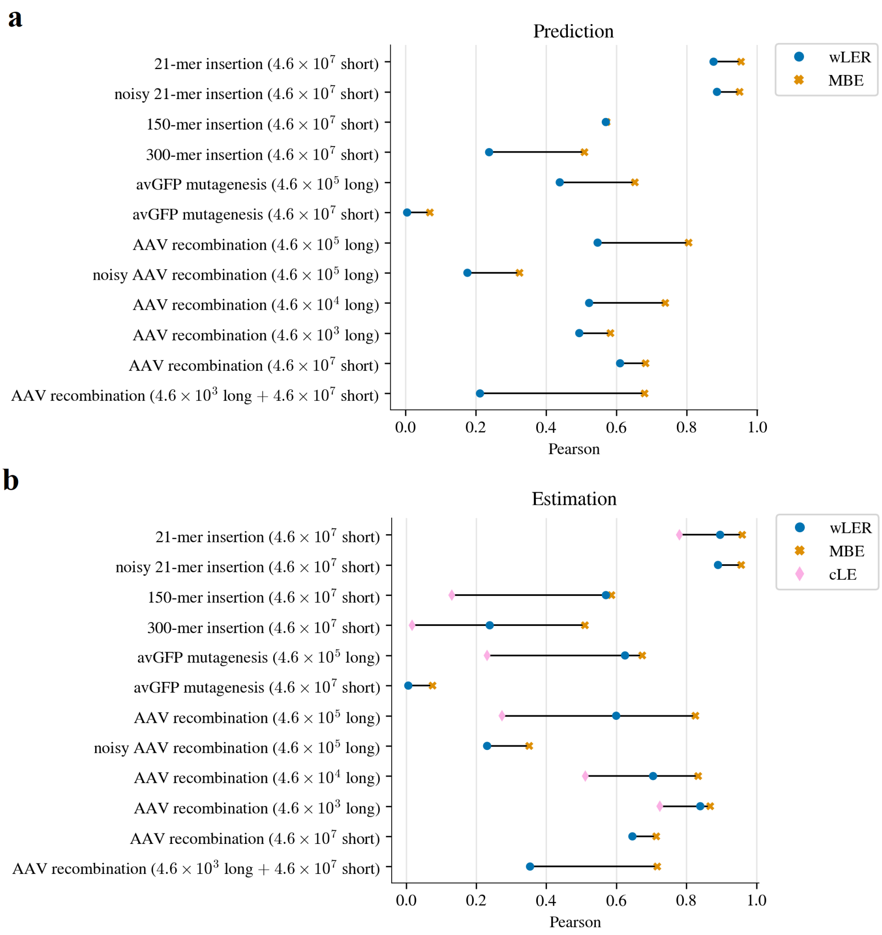

Fig. S2 Simulation results evaluated using Pearson correlation. (a) and (b) are the same as Fig. 4a and b, respectively, but display the Pearson correlation between model predictions and ground truth fitness.

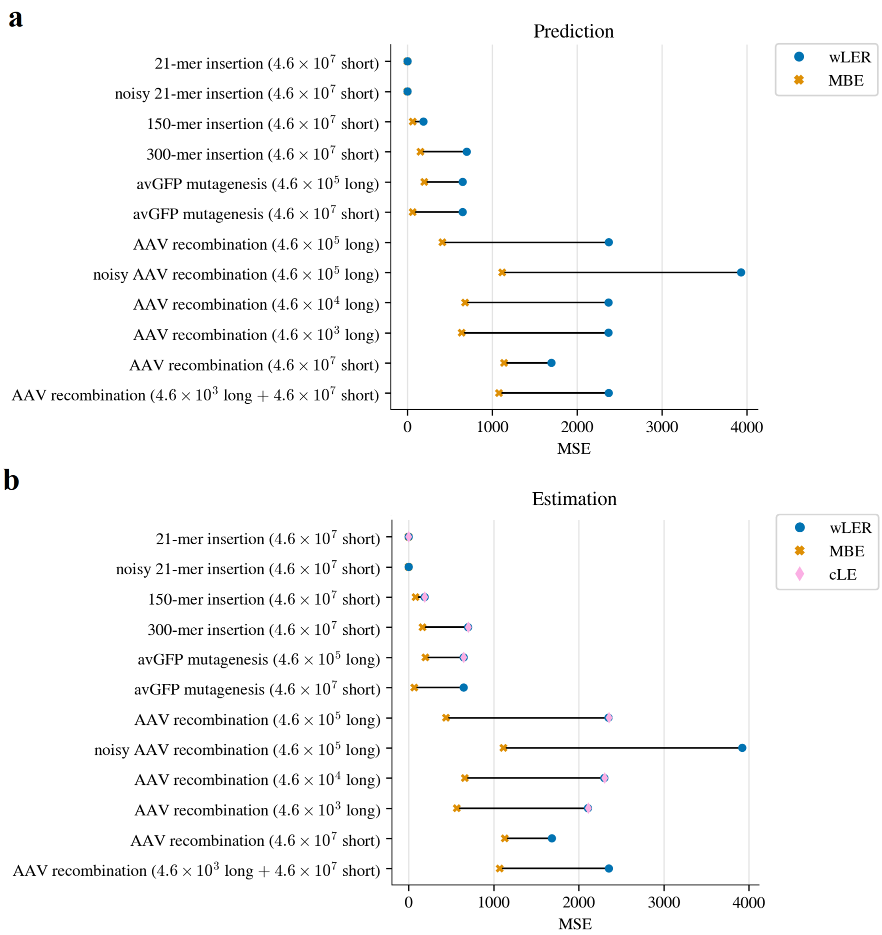

Fig. S3 Simulation results evaluated using mean squared error. (a) and (b) are the same as Fig. 4a and b, respectively, but display the mean squared error between model predictions and ground truth fitness.

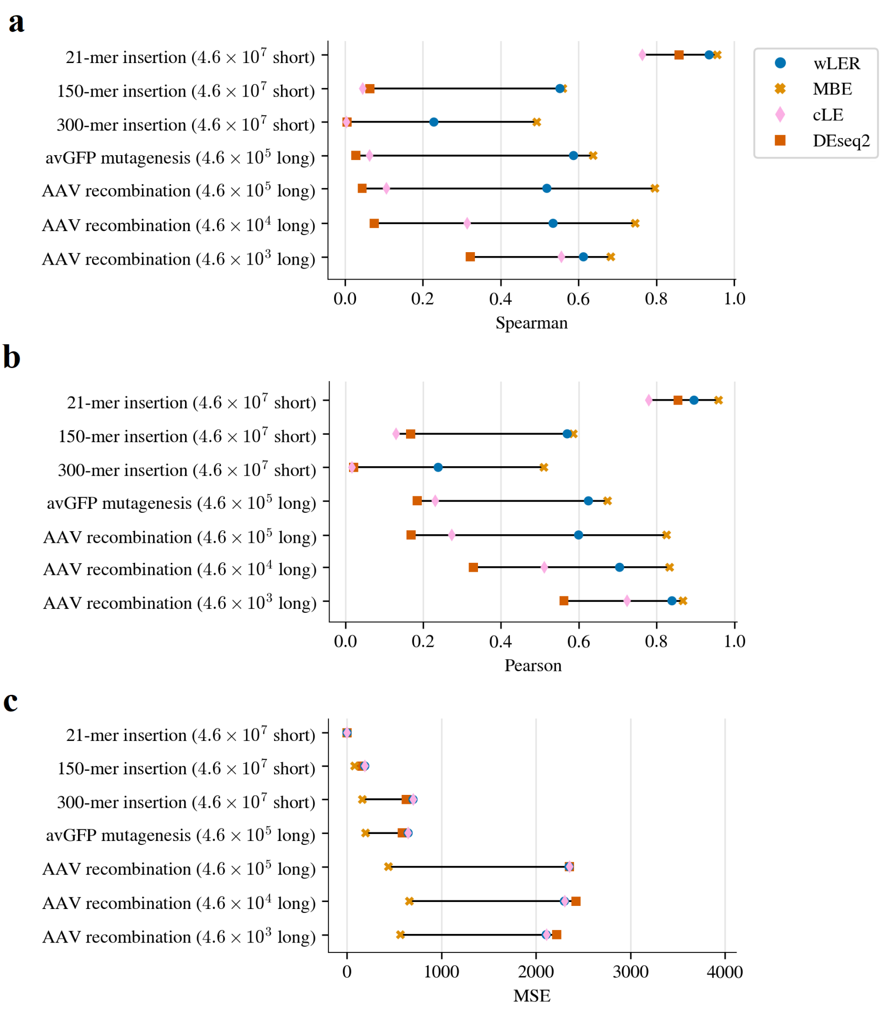

Fig. S4 Simulation results compared to DEseq2. (a) Spearman correlation, (b) Pearson correlation, and (c) mean squared error between ground truth log-enrichment and cLE, wLER, MBE, and DEseq2 log-enrichment estimates. For wLER and MBE, each panel displays the performance achieved by the best-performing model architecture for each method on each simulated dataset. All methods use a single replicate of sequencing data except for DEseq2 which requires multiple replicates. DEseq2 results are obtained using three replicates which differ in the random seed that was used to sample read counts from the ground truth fitness. Note that DEseq2 is designed to be used with multiple biological replicates per condition, which may be of a different nature than these simulated replicates.

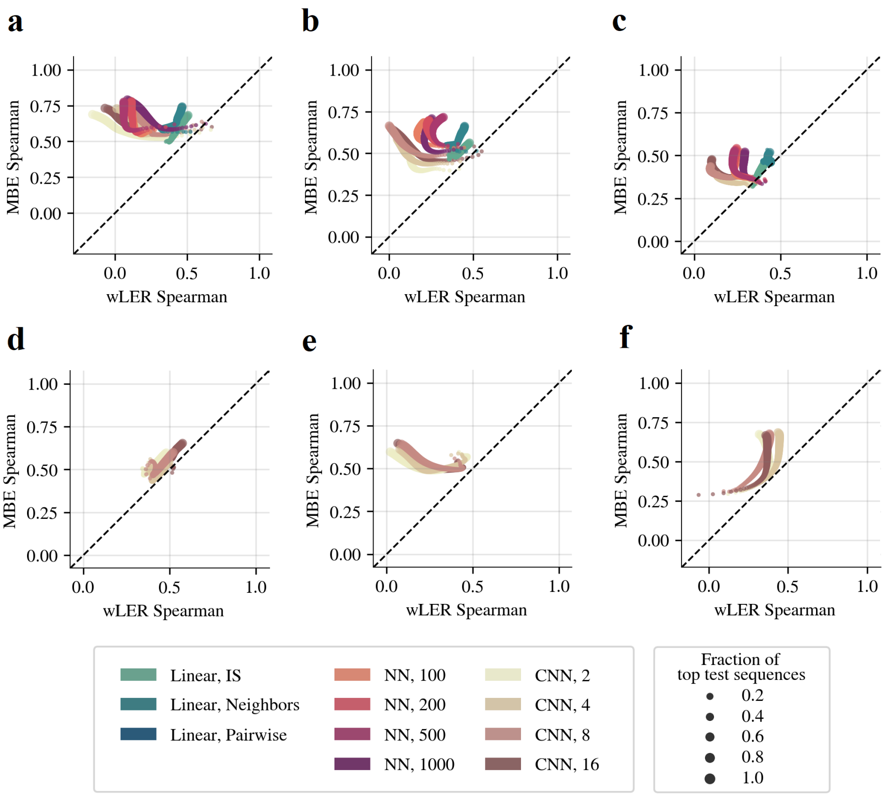

Fig. S5 Simulated library results with increasing long read sparsity and short reads. Compares Spearman correlation between simulated ground truth fitness and wLER or MBE predictions on held-out sequences of interest when models are trained using the simulated AAV recombination datasets with (a) 4.6×10^5 long reads, (b) 4.6×10^4 long reads, (c) 4.6×10^3 long reads, (d) 4.6×10^7 short reads, and (e) a combination of 4.6×10^3 long and 4.6×10^7 short reads, and (f) the avGFP mutagenesis dataset with 4.6×10^7 short reads. Each panel compares the Spearman correlation achieved by the wLER and MBE approaches using the same model architecture and hyper-parameters. Dot size represents the fraction of test sequences with highest ground truth fitness used to compute Spearman correlation. Only CNNs are included in d-f since the linear and NN models cannot operate on variable-length sequences.

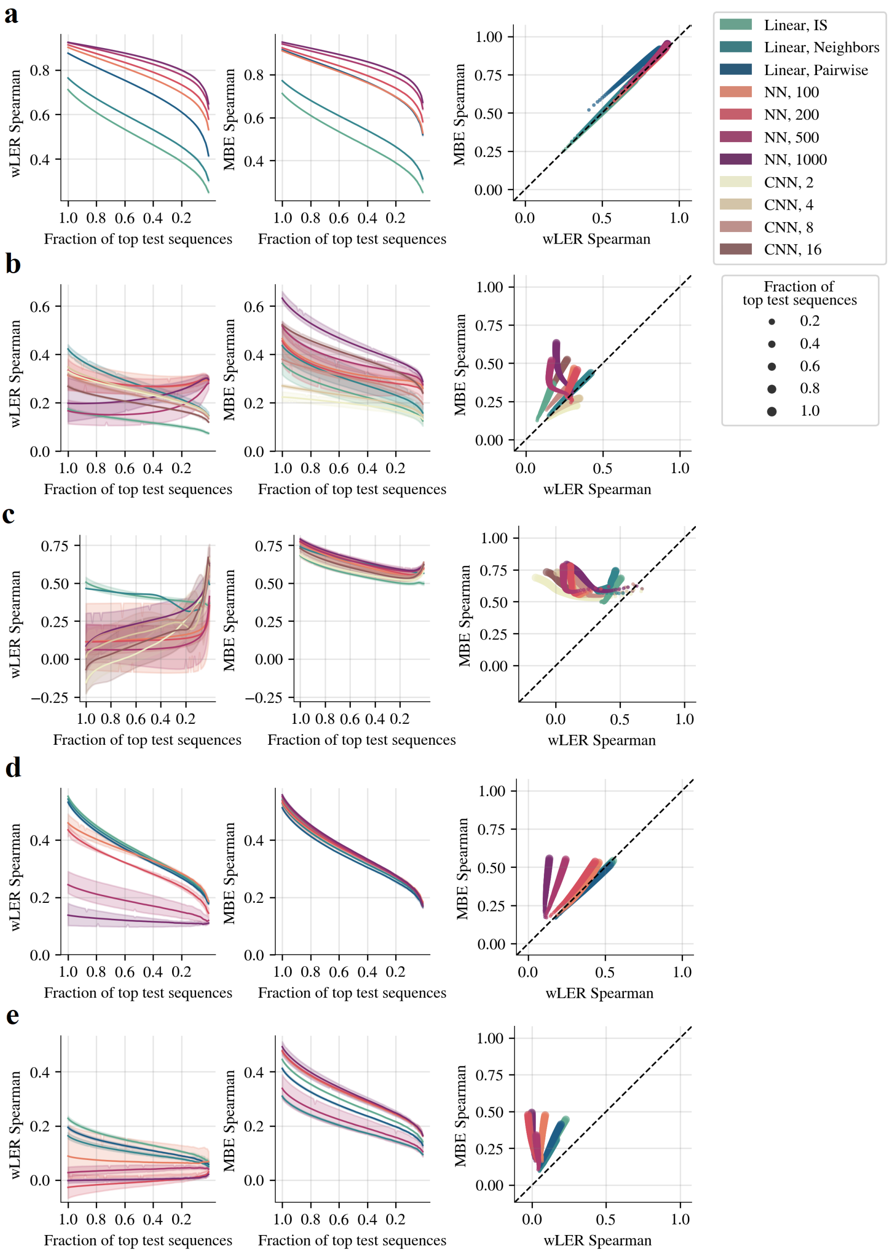

Fig. S6 Generalized Spearman for simulated library prediction. Comparison of generalized Spearman correlation between simulated ground truth fitness and wLER or MBE predictions on held-out full-length sequences for the simulated (a) 21-mer insertion (4.6×10^7 short reads), (b) avGFP mutagenesis (4.6×10^5 long reads), (c) AAV recombination (4.6×10^5 long reads), (d) 150-mer insertion (4.6×10^7 short reads), and (e) 300-mer insertion (4.6×10^7 short reads) datasets. In each row, the leftmost panel compares the performance of wLER for each model architecture (the horizontal axis displays the fraction of top test sequences with highest ground truth fitness used to calculate Spearman correlation), the center panel is the same as the left panel MBE, and the rightmost panel is a paired plot version of the left and center panels (dot size represents the fraction of top test sequences used to compute Spearman correlation).

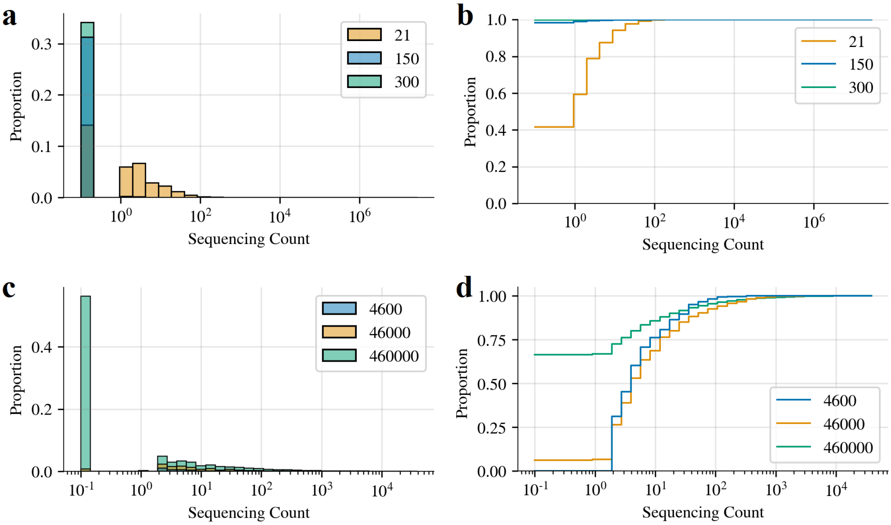

Fig. S7 Sequencing count histograms for simulated insertion and recombination libraries. Histogram (left) and cumulative histogram (right) of simulated post-selection sequencing counts for the (a-b) 21-mer, 150-mer, and 300-mer insertion datasets, and (c-d) AAV recombination datasets with 4.6×10^5, 4.6×10^4, and 4.6×10^3 long reads.

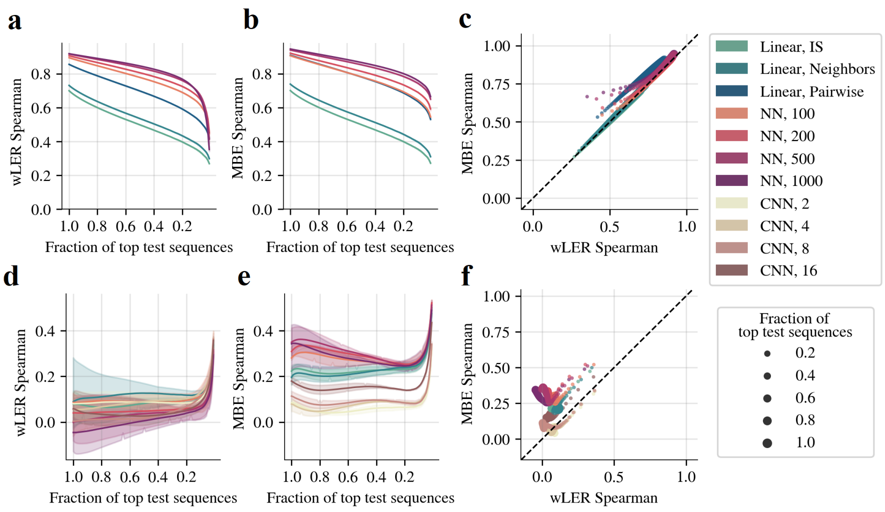

Fig. S8 Generalized Spearman for prediction with simulated sequencing errors. Comparison of the Spearman correlation between simulated ground truth fitness and wLER or MBE predictions on held-out full-length library sequences when models are trained using the simulated (a-c) noisy 21-mer insertion (4.6×10^7 short reads) and (d-f) noisy AAV recombination (4.6×10^5 long) datasets. The noisy 21-mer insertion dataset includes substitution errors added to the training set at a uniform error rate of 0.1%, consistent with Illumina's next-generation sequencers [11]. The noisy AAV recombination dataset contains simulated PacBio SMRT sequencing errors added to the training set using SimLoRD [12]. In each row, the leftmost panel compares the performance of wLER for each model architecture (the horizontal axis displays what fraction of top test sequences with highest ground truth fitness is used to calculate Spearman correlation), the center panel is the same as the left panel for MBE, and the rightmost panel is a paired plot version of the left and center plots (dot size represents the fraction of top test sequences used to compute Spearman correlation).

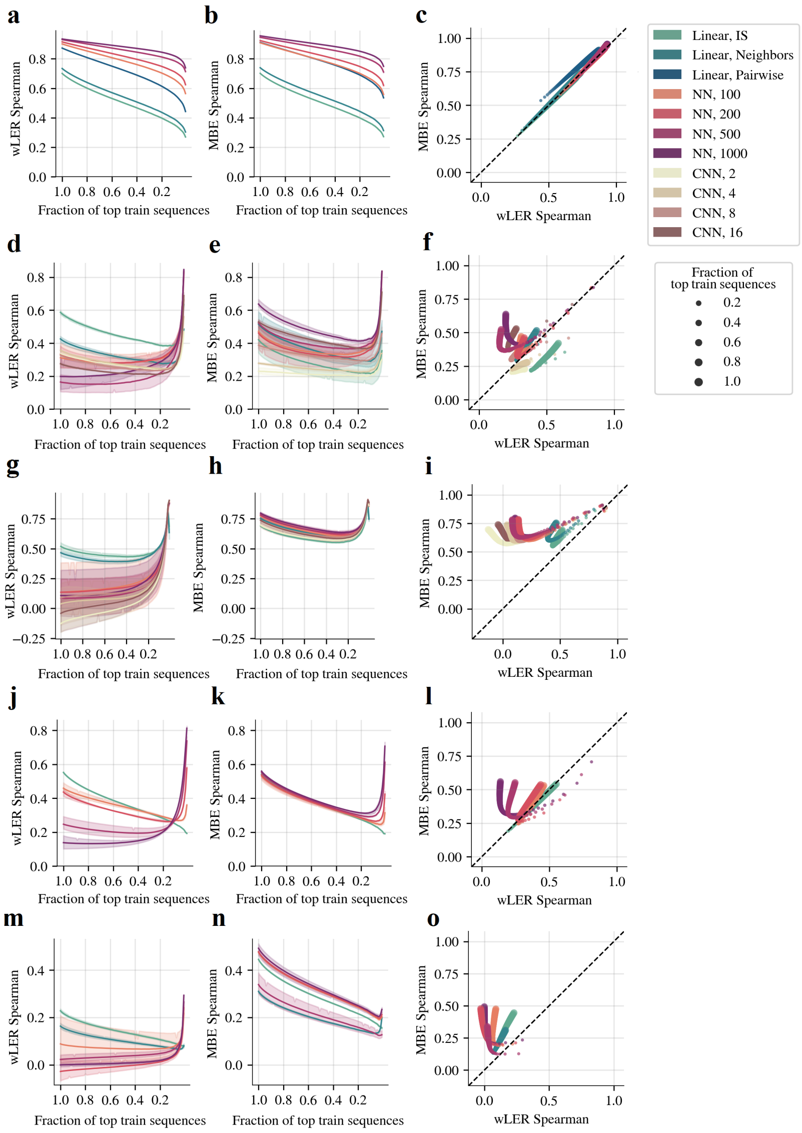

Fig. S9 Generalized Spearman for simulated library estimation. Comparison of the Spearman correlation between simulated ground truth fitness and wLER or MBE LE estimates for full-length library sequences observed during training for the simulated (a-c) 21-mer insertion (4.6×10^7 short reads), (d-f) avGFP mutagenesis (4.6×10^5 long reads), (g-i) AAV recombination (4.6×10^5 long reads), (j-l) 150-mer insertion (4.6×10^7 short reads), and (m-o) 300-mer insertion (4.6×10^7 short reads) datasets. In each row, the leftmost panel compares the performance of wLER for each model architecture (the horizontal axis displays what fraction of top test sequences with highest ground truth fitness is used to calculate Spearman correlation), the center panel is the same as the leftmost panel for MBE, and the rightmost panel is a paired plot version of the left and center plots (dot size represents the fraction of top test sequences used to compute Spearman correlation).

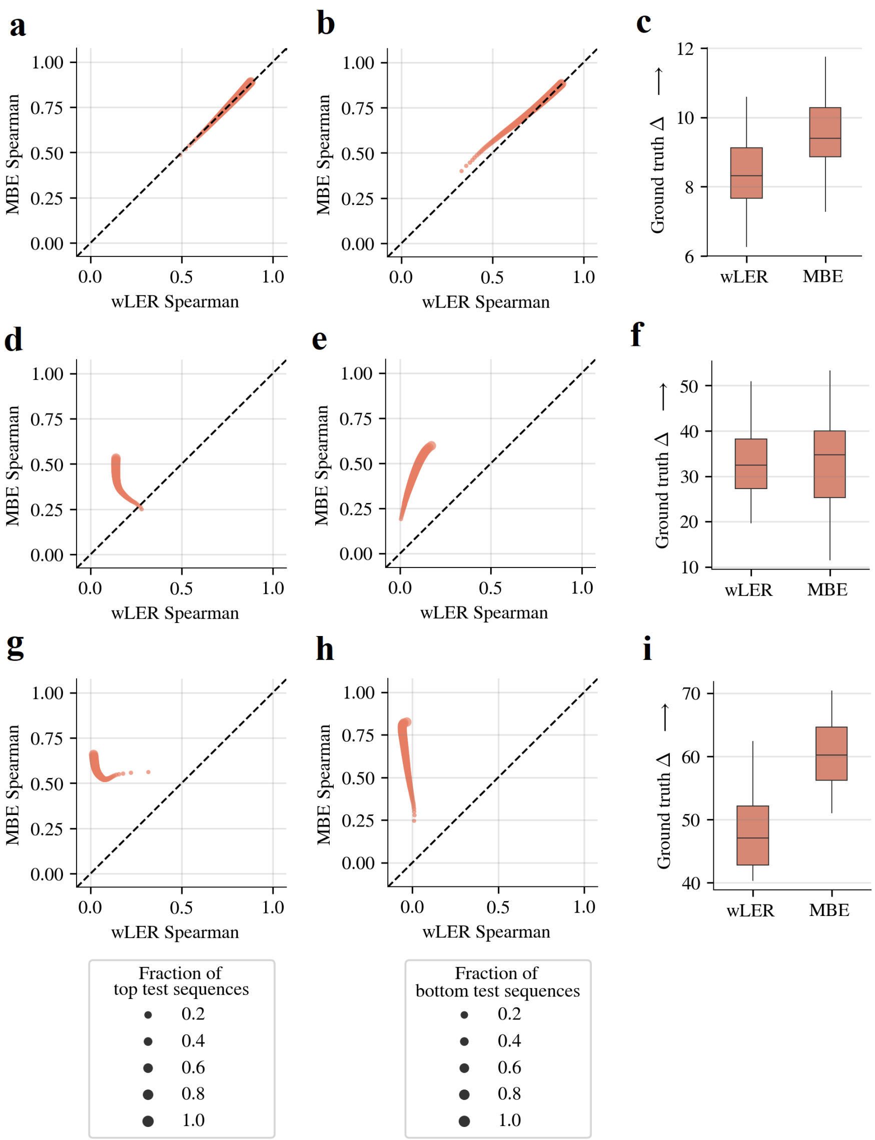

Fig. S10 Simulated positive, negative, and selectivity selection results. Comparison of wLER and MBE on (left) prediction for sequences with high ground truth positive fitness, (center) prediction for sequences with low ground truth negative fitness, and (right) selection for sequence selectivity for the simulated (a-c) 21-mer insertion (4.6×10^7 short reads), (d-f) avGFP mutagenesis (4.6×10^5 long reads), and (g-i) AAV recombination (4.6×10^5 long reads) datasets. For positive fitness, dot size represents the fraction of top test sequences according to highest ground truth positive fitness. For negative fitness, dot size represents the fraction of test sequences with lowest ground truth negative fitness. In each row, the rightmost panel displays ground truth selectivity (the difference between positive and negative fitness values, Δ) for the top ten test sequences according to each model's predicted selectivity (the difference between predicted fitness values) for each of the three test folds.

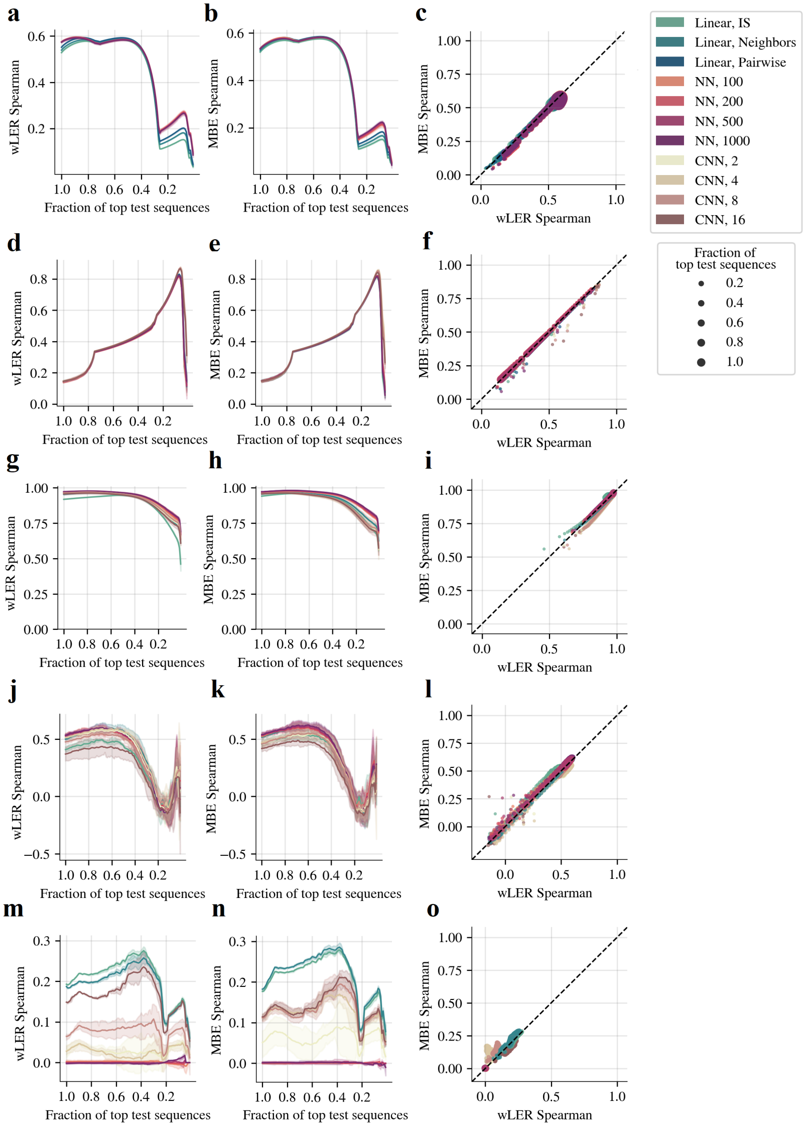

Fig. S11 Experimental library cross-validation results. Comparison of the Spearman correlation between wLER or MBE predictions and observed cLE estimates on the real sequencing datasets from (a-c) the AAV5 insertion library from Zhu *et al.* [1], (d-f) the SARS-CoV-2 tiled peptide library from Huisman *et al.* [13], (g-i) the GB1 double site saturation mutagenesis library from Olson *et al.* [14], (j-l) the library of natural and designed chorismate mutase homologs from Russ *et al.* [15], and (m-o) the Bgl3 random mutagenesis library from Romero *et al.* [16]. In each row, the leftmost panel compares the performance of wLER for each model architecture restricted to a given top fraction of held-out test sequences with highest observed cLE estimate, the center panel is the same as the leftmost panel for MBE, and the rightmost panel is a paired plot version of the left and center panels (dot size represents the fraction of top test sequences with highest observed cLE estimate used to compute Spearman correlation).

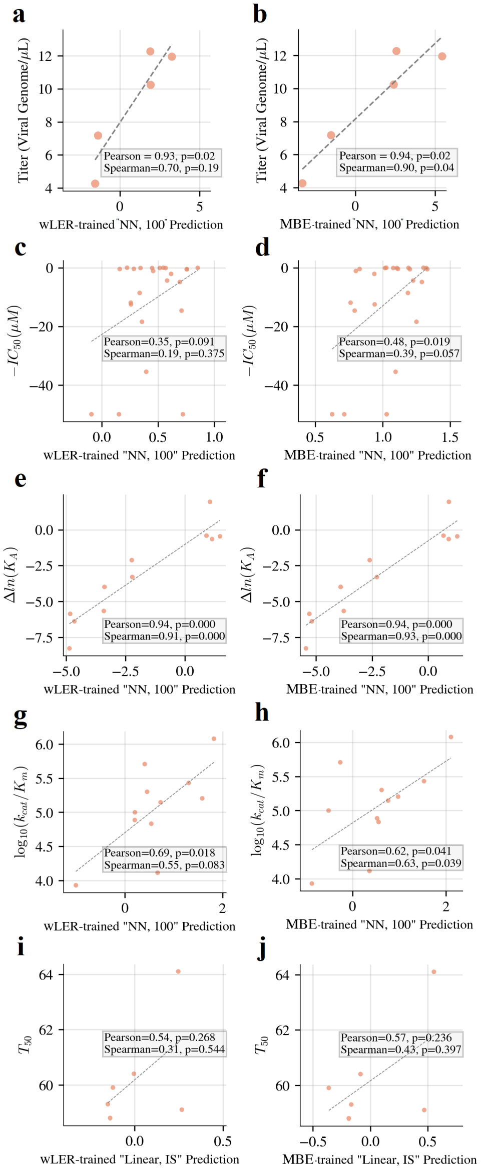

Fig. S12 Low-throughput experimental property measurement predictions. Comparison of (left) wLER and (right) MBE predictions and experimental property measurements from (a-b) Zhu *et al.* [1] (packaging titer), (c-d) Huisman *et al.* [13] (IC_{50}, half maximal inhibitory concentration), (e-f) Olson *et al.* [14] ($\Delta\ln(K_A)$, change in log-binding constant), (g-h) Russ *et al.* [15] ($\log_{10}(k_{cat}/K_m)$, log-second-order reaction rate constant), and (i-j) Romero *et al.* [16] (T_{50}, temperature where half of the protein is inactivated in ten minutes). The 100-unit NN architecture is used for all datasets except that from Romero *et al.* [16] for which the linear architecture with IS features is used.

Table S1 Comparison of Spearman correlation between experimental IC_{50} measurements from Huisman *et al.* [13] and wLER predictions, MBE predictions, or reported NetMHCIIpan4.0 predictions from Huisman *et al.* [13]. The 100-unit NN architecture is used for the wLER and MBE methods.

	Spearman	p-value
MBE	0.394	0.057
wLER	0.190	0.375
NetMHCIIpan4.0 %Rank	0.275	0.193

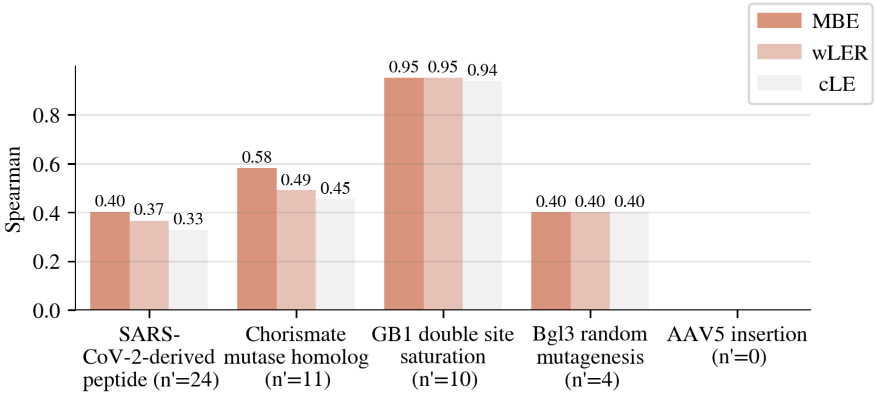

Fig. S13 Real experimental estimation results. Comparison of Spearman correlation between wLER, MBE, or cLE estimates and experimental property measurements from the SARS-CoV-2-derived peptide [13], Chorismate mutase homolog [15], GB1 double site saturation [14], Bgl3 random mutagenesis [16], and AAV5 insertion [1] libraries. Each method was trained on real pre- and post-selection sequencing data, then used to predict the fitness of the n' experimentally-measured sequences that are also appeared in the corresponding high-throughput sequencing data during training. In contrast to the prediction task (Fig. 7), to evaluate the estimation task, we could only evaluate on sequences that overlapped between the high- and low-throughput experiments for each of the five protein datasets. This had the effect of changing the already tiny test set sample sizes from $n = 5$ to $n' = 0$ (AAV5), $n = 6$ to $n' = 4$ (Bgl3), $n = 11$ to $n' = 10$ (Chorismate mutase), and to leave the remaining two test sets the same size ($n = n' = 11$ for GB1 and $n = n' = 24$ for SARS-CoV-2). Unsurprisingly, the overall p-value for the combined test data across all datasets (now with 49 instead of 57 test sequences), was not statistically significant ($p = 0.237$). However, based on our simulations, we expect that with a comparable amount of test data, the estimation results improvement would have been significant, as for the prediction task. The 100-unit NN model architecture was used for all datasets except the Bgl3 dataset, for which the linear architecture with Independent Sites features was used.

References

[1] Zhu, D., Brookes, D.H., Busia, A., Carneiro, A., Fannjiang, C., Popova, G., Shin, D., Chang, E.F., Nowakowski, T.J., Listgarten, J., et al.: Machine learning-based library design improves packaging and diversity of adeno-associated virus (aav) libraries. bioRxiv (2021)

[2] Sugiyama, M., Suzuki, T., Kanamori, T.: Density Ratio Estimation in Machine Learning. Cambridge University Press, ??? (2012)

[3] Qin, J.: Inferences for case-control and semiparametric two-sample density ratio models. Biometrika **85**(3), 619–630 (1998)

[4] Sugiyama, M., Suzuki, T., Kanamori, T.: Density-ratio matching under the bregman divergence: a unified framework of density-ratio estimation. Annals of the Institute of Statistical Mathematics **64**(5), 1009–1044 (2012)

[5] Newey, W.K., McFadden, D.: Large sample estimation and hypothesis testing. Handbook of econometrics **4**, 2111–2245 (1994)

[6] Bryant, D.H., Bashir, A., Sinai, S., Jain, N.K., Ogden, P.J., Riley, P.F., Church, G.M., Colwell, L.J., Kelsic, E.D.: Deep diversification of an aav capsid protein by machine learning. Nature Biotechnology **39**(6), 691–696 (2021)

[7] Poelwijk, F.J., Socolich, M., Ranganathan, R.: Learning the pattern of epistasis linking genotype and phenotype in a protein. Nature communications **10**(1), 1–11 (2019)

[8] Gneiting, T., Balabdaoui, F., Raftery, A.E.: Probabilistic forecasts, calibration and sharpness. Journal of the Royal Statistical Society Series B: Statistical Methodology **69**(2), 243–268 (2007)

[9] Niculescu-Mizil, A., Caruana, R.: Predicting good probabilities with supervised learning. In: Proceedings of the 22nd International Conference on Machine Learning, pp. 625–632 (2005)

[10] Guo, C., Pleiss, G., Sun, Y., Weinberger, K.Q.: On calibration of modern neural networks. In: International Conference on Machine Learning, pp. 1321–1330 (2017). PMLR

[11] Fox, E.J., Reid-Bayliss, K.S., Emond, M.J., Loeb, L.A.: Accuracy of next generation sequencing platforms. Next generation, sequencing & applications **1** (2014)

[12] Stöcker, B.K., Köster, J., Rahmann, S.: Simlord: simulation of long read

data. Bioinformatics **32**(17), 2704–2706 (2016)

[13] Huisman, B.D., Dai, Z., Gifford, D.K., Birnbaum, M.E.: A high-throughput yeast display approach to profile pathogen proteomes for mhc-ii binding. eLife **11**, 78589 (2022). https://doi.org/10.7554/eLife.78589

[14] Olson, C.A., Wu, N.C., Sun, R.: A comprehensive biophysical description of pairwise epistasis throughout an entire protein domain. Current biology **24**(22), 2643–2651 (2014)

[15] Russ, W.P., Figliuzzi, M., Stocker, C., Barrat-Charlaix, P., Socolich, M., Kast, P., Hilvert, D., Monasson, R., Cocco, S., Weigt, M., *et al.*: An evolution-based model for designing chorismate mutase enzymes. Science **369**(6502), 440–445 (2020)

[16] Romero, P.A., Tran, T.M., Abate, A.R.: Dissecting enzyme function with microfluidic-based deep mutational scanning. Proceedings of the National Academy of Sciences **112**(23), 7159–7164 (2015)
