# Supplementary material for: MBE: model-based enrichment estimation and prediction for differential sequencing data
Source: Genome Biol. 2023 Oct 2;24:218. doi: 10.1186/s13059-023-03058-w (PMC10544408; doi:10.1186/s13059-023-03058-w)
Supplement: Supplementary file 2 — Additional file 2. Review history. [file 13059_2023_3058_MOESM2_ESM.docx]

**1^st^ round**

**Reviewer 1**

The authors presented a machine learning framework for differential sequence analysis that purportedly fills a methodological gap. Perhaps this work is interesting, but I have the following questions that I hope will help improve the quality of the manuscript.

##Major comments:

1) I think the proposed method, model-based enrichment, has the potential to be extensively utilized in various applications, including antibody design, enhancing thermostability, and improving the packaging efficiency or infectivity of viral vectors. However, overall the motivation of this work is not very clear and I find the current description somewhat confusing in several sentences. For instance, it is unclear what specific "two conditions" are being referred to; in the last paragraph of Background, it is unclear what specific "desired properties" are being referred to. Could you please provide clarification on what is meant by these in this main text?

2) Limitations of log-enrichment estimates that may cause a loss of important available information in a number of practical settings. Why the 'hybrid reads' is a fundamental shortcoming setting? Could you please provide clarification on what is meant by " hybrid reads " in this context? My suggestion is to explain more details about each setting that why these settings are shortcoming settings.

3) The competing method wLER presented by Zhu et al. to model the log_ei using the weighted LE regression, while MBE is to model the density ratio estimation problem. It looks like MBE is the logistic version of LE regression while wLER is a Guassian version of LE regression if I understand correctly. What I am concerned about is why the MBE could overall outperform the wLER? Does it depend on the evaluation methods, how about Pearson correlation?

4) MBE was explored to deal with the settings with more than two conditions. However, only two conditions were considered in the simulation studies, it is necessary to add additional simulation studies to demonstrate the ability of such a scenario. In addition, could you display the details of the modeling of a case for more than two conditions?

5) The authors declared that MBE is designed for quantifying changes in sequence abundances and learning models to predict such differences for unobserved sequences. However, all real data application results are used to demonstrate the ability of prediction. Can the author also show the sequence abundance quantification ability for all methods? Besides, since the authors used the NN and CNN architectures to estimate the parameters in the model, is it possible for MBE to output p-values to assess the significance of changes in sequence abundance between conditions?

6) The authors applied MBE on real data sets with all model architectures and hyper-parameters and then selected the best combination. Is there a straightforward guide for users to select appropriate model architectures and hyper-parameters under different conditions?

7) This method is potentially a valuable toolkit for differential sequence analysis. If the authors have confidence in the performance of the proposed method, this would be greatly facilitated by providing a ready-made implementation in the form of an R or Python package; The lack of implementation will severely limit the practical value of the proposed method. In the present submission, only the source code is provided and a general-purpose implementation is missing.

##Minor comments:

1) The page 14 line 601, "the are" might be "there are".

2) I think it would be helpful to have a simple diagram explaining how you get your features as input into your model. If I understand your modeling correctly, MBE models' total reads and mapped reads directly estimate the corresponding underlined enrichment probability between two conditions. A diagram would be helpful for understanding the MBE models and "pre, post-selection sequencing data".

3) How can we distinguish the different fractions of lowest test sequences in sub-figures?, for example a, b, etc.

**Reviewer 2**

The authors describe a novel approach for differential sequence analysis including quantifying changes in sequence abundances and making enrichment predictions for unobserved sequences. The approach is machine learning based, termed model-based enrichment (MBE), and proposes to overcome limitations of the existing methods (count-based log-enrichment and weighted log-enrichment regression (wLER)) by sharing information across non-identical reads. The authors perform a thorough performance comparison, particularly for predictions of unobserved sequences, by simulating datasets representing several different conditions (experimental design, sequencing data type etc.) and confirm the performance using real datasets.

The new analysis approach appears superior to the existing methods, and should be of benefit in sequence prediction experiments.

Major comments:
1. Given the various comparisons of models and datasets and made in this paper, it would be beneficial to have an overall schematic of the study design as well as visual representation of the proposed analysis approach as the first figure.
2. I believe the paper would benefit from rearranging some text and sections to improve readability - for example, introducing datasets (e.g real experimental data) directly before the performance results to prevent the reader from jumping back and forth.
3. Since the focus of this paper was to introduce a new analysis approach for other researchers to use, the code should be packaged as a library and/or should have clearly defined instructions for implementation, with practical examples.
4. The real experimental datasets were all short-read based, are there any long-read or hybrid (long + short read) datasets that could also be included for confirmation of performance? Particularly since MBE showed the biggest performance advantage against wLER in these settings.
5. While the main motivation for this approach was to make enrichment predictions for unobserved sequences, the authors also touch on a more classical analysis of estimation of log-enrichment. For this second case, as I understood it, it was an alternative for count-based enrichment analysis, e.g. for RNA-seq. If this is correct, should this approach not be further assessed against popular negative binomial modelling methods, such as DEseq2 or edgeR? Alternatively, should the focus be reframed to only consider predictions for unobserved sequences?

Minor comments:
1. Illumina and Pacbio sequencing error rates are estimated around 0.1%, not 1% (however, Nanopore long read error rates are around 1%). This should be corrected in the text.
2. Could the authors please re-order and group simulated experiments in figure 1 and extended data figure 3 in some logical way, perhaps following the order in table 1 (different length insertions, short vs long avGFP mutagenesis, AAV - different sparseness of long reads, short reads and hybrid experiment)?
3. Could the authors please use consistent names for datasets in figure 4 as listed in table 2?
4. In figure 4, alongside Spearman correlation, it would also be helpful to plot the individual correlations of predictions vs functional validation per sequence.

**Authors’ response**

Reviewer #1:

1) I think the proposed method, model-based enrichment, has the potential to be extensively utilized in various applications, including antibody design, enhancing thermostability, and improving the packaging efficiency or infectivity of viral vectors. However, overall the motivation of this work is not very clear and I find the current description somewhat confusing in several sentences. For instance, it is unclear what specific "two conditions" are being referred to; in the last paragraph of Background, it is unclear what specific "desired properties" are being referred to. Could you please provide clarification on what is meant by these in this main text?

Thanks for highlighting this lack of clarity, which we have now addressed by revising and augmenting text in the manuscript (lines 064 and 113).

2) Limitations of log-enrichment estimates that may cause a loss of important available information in a number of practical settings. Why the 'hybrid reads' is a fundamental shortcoming setting? Could you please provide clarification on what is meant by " hybrid reads " in this context? My suggestion is to explain more details about each setting that why these settings are shortcoming settings.

Again, thanks for this valuable feedback on lack of clarity, which we have mitigated by introducing a new figure (Fig. 1) illustrating each of the fundamental shortcoming settings, including that of hybrid reads. We have also added further clarifications in the text of the manuscript (lines 181-245).

3) The competing method wLER presented by Zhu et al. to model the log_ei using the weighted LE regression, while MBE is to model the density ratio estimation problem. It looks like MBE is the logistic version of LE regression while wLER is a Guassian version of LE regression if I understand correctly. What I am concerned about is why the MBE could overall outperform the wLER? Does it depend on the evaluation methods, how about Pearson correlation?

Ah, yes, this was not as clear as it could have been. We have tried to clarify throughout the text (lines 496, 1161 and 1181), and also wrote a new section to explain this better, copied here because of the importance:

Contrasting wLER and MBE

At first glance, it may appear that the main difference between wLER and MBE is that the former uses regression, while the latter uses classification. However, conceptually, the more important difference is that wLER (and any regression-based LE method) uses cLE estimates as regression labels. Hence, wLER is dependent on cLE estimation and consequently is affected by all the limitations of cLE described earlier—namely those arising from the inability to share information across similar, but distinct reads. In contrast, MBE is trained using the raw reads directly, labeling each as coming from one of two conditions, thereby bypassing the issues arising from cLE estimates. That is, MBE’s classification labels need not be estimated, but are given to us for free, with 100% accuracy. It is this distinction that lies at the core of why MBE tends to outperform wLER. That one method uses classification instead of regression is a byproduct of the technical underpinnings used to achieve this desideratum.

As for whether our results depend on if Spearman rather than Pearson correlation is used, we have now added new results that report the Pearson correlation and mean squared error (MSE) for all simulation results (Extended Data Figures 2 and 3), pointed to on line 1074. These results show that our overall conclusions remain unchanged:

MBE outperforms wLER on all the simulated datasets. We note that the reason we did not originally include such metrics is that, when it comes to the real data rather than simulations, the experimental property measurements are not in units of log- enrichment (or equivalently, density ratios), rather, they are in units (e.g., molar concentration or degrees Celsius) that we can expect only to be monotonically (but not necessarily linearly) related to the log-enrichment/density ratio. Hence, the only reasonable correlation to use for these is a rank-based correlation such as Spearman, rather than Pearson which assumes a linear relationship. For this reason, we only use Spearman correlation for reporting results on the real data, now noted on line 958 in the manuscript.

4) MBE was explored to deal with the settings with more than two conditions. However, only two conditions were considered in the simulation studies, it is necessary to add additional simulation studies to demonstrate the ability of such a scenario. In addition, could you display the details of the modeling of a case for more than two conditions?

Once more, thanks for highlighting this lack of clarity. We have now added a new section Generalization of wLER and MBE to more than two conditions (line 613) explaining how each method can be generalized to multiple conditions. We have also added details on the multi-condition setting to A new approach: model-based enrichment (line 534), Overview of simulation experiments (line 837), and Model architectures (line 890).

As for adding new experiments to correspond to the multi-condition setting, it turns out we had already done so, but did not clearly explain this as it was accidentally hidden under the guise of our selectivity experiments—those where the motivating goal was to be able to engineer a protein that scores highly for one property (the positive selection) and scores low on a different property (the negative selection)—hence the three conditions are i) the baseline starting library, ii) the positively selected library, and iii) a negatively selected library. We have now made this more explicit in the manuscript (lines 362, 837, 889, and 1235).

5) The authors declared that MBE is designed for quantifying changes in sequence abundances and learning models to predict such differences for unobserved sequences. However, all real data application results are used to demonstrate the ability of prediction. Can the author also show the sequence abundance quantification ability for all methods?

We have now included these new results, but first, let us explain why they were not there in the first place. Recall that the way we conducted the real data prediction task experiments was to: for each of the five protein datasets, i) train a MBE/wLER model on high-throughput sequencing data, and then ii) use that model to make test predictions on the low-throughput biophysical assay data. This procedure did not require that there was any overlap in sequences between the low and high-throughput data because prediction is meant to be on new sequences not seen in the high- throughput data during train time (in fact, we removed those that did overlap between sources from the high-throughput sequencing data before training to ensure this was so). In contrast, to evaluate the abundance quantification (aka. estimation) task, we could only test on sequences that did overlap between the high-throughput and low- throughput experiments for each of the five protein data sets. This had the effect of changing the already tiny test set sample sizes from 5 to 0 (AAV5), 6 to 4 (Bgl3), 11 to 10 (Chorismate mutase) and to leave the remaining two data sets of the same size (GB1 with 11, and SARS-CoV-2 with 24). Nevertheless, the results for these (still less well-powered) abundance quantification evaluations now appear in Extended Data Figure 13, and are pointed to on line 1464. Although the quantitative differences between methods are not as striking as when evaluating the prediction task, we see that the trend remains similar—MBE performs maximally, sometimes beating the other methods (when test sample size was the same as in prediction), and sometimes tied with them (when sample size was smaller), but MBE never performed worse than the other methods. Unsurprisingly, the overall p-value for the combined test data across all datasets (now with 49 instead of 57 test points), was not statistically significant (p=0.24). However, based on our simulations, we expect that with a comparable amount of test data, the estimation results improvement would have been significant, as on the prediction task. These details have been included in the caption of Extended Data Figure 13.

Besides, since the authors used the NN and CNN architectures to estimate the parameters in the model, is it possible for MBE to output p-values to assess the significance of changes in sequence abundance between conditions?

We’re not certain what the reviewer had in mind, but because p-values are typically associated with statistical hypothesis testing rather than predictions from machine learning models, it is not clear to us how one would do so, but would welcome suggestions from the reviewer. However, to elaborate, our point of view is as follows. A p-value from a hypothesis test—which measures the probability the observed test statistic occurred randomly given a specific null hypothesis—is fundamentally different from uncertainty estimates often associated with machine learning predictions (e.g., a confidence interval/set, or noise estimation for regression; probability for each class in classification). In particular, a probabilistic classifier’s output probability for each class can be interpreted as the model’s confidence in its prediction; further, when the model is calibrated [1-3], this confidence has particular technical guarantees. While these guarantees are different from p-values, they serve a similar purpose by acting as a human-interpretable metric of confidence for a particular prediction. Although the question of how best to calibrate modern model architectures for probabilistic classification is outside the scope of our manuscript, there are many existing and well- explored approaches for calibrating classifiers—including feed-forward and convolutional neural network classifiers [1]—which could be used to produce a calibrated classifier for use the MBE approach, if desired for the specific application. We now discuss these points in the manuscript (Statistical guarantees for model-based enrichment, line 2991).

6) The authors applied MBE on real data sets with all model architectures and hyper- parameters and then selected the best combination. Is there a straightforward guide for users to select appropriate model architectures and hyper-parameters under different conditions?

Although selecting and training classifier architectures may require some familiarity with working with machine learning models, one advantage of the MBE approach is that one can use standard cross-validation for model and hyper-parameter selection because the underlying modeling problem is a standard supervised classification problem [4]. In practice, one would ideally tune a classifier on one’s specific dataset using standard software packages for training and tuning machine learning classification models that allow one to search over a broad range of hyper-parameter settings to identify a combination that works best according to validation loss on the specific dataset in question. Based on your feedback, we have now added this short discussion to the manuscript (lines 512 and 875).

7) This method is potentially a valuable toolkit for differential sequence analysis. If the authors have confidence in the performance of the proposed method, this would be greatly facilitated by providing a ready-made implementation in the form of an R or Python package; The lack of implementation will severely limit the practical value of the proposed method. In the present submission, only the source code is provided and a general-purpose implementation is missing.

Agreed. We have now created a ‘model_based_enrichment’ Python package which has been uploaded with the revision, and will be publicly released upon acceptance. Our package provides a general-purpose implementation of MBE to be used on a dataset of sequences and corresponding sequencing counts, for two or more conditions. The package can readily use any classifier in scikit-learn [8], one of the most widely-used, and easy-to-use machine learning libraries for Python, for which we provide clear demonstrations. We also include an example of how one can go beyond scikit-learn; namely, we show how to use any TensorFlow classifier. In particular, we provide a clear example demonstrating how to use MBE with the specific model architectures appearing in our manuscript.

##Minor comments:

1) The page 14 line 601, "the are" might be "there are".

Corrected.

2) I think it would be helpful to have a simple diagram explaining how you get your features as input into your model. If I understand your modeling correctly, MBE models' total reads and mapped reads directly estimate the corresponding underlined enrichment probability between two conditions. A diagram would be helpful for understanding the MBE models and "pre, post-selection sequencing data".

We hope that our newly added Figure 2, and the corresponding text in that figure caption, clear up this issue. We note that in our manuscript, we do not use any mapped reads, although we mention this possibility in the Discussion (line 1559). Moreover, in the simulated experiments, we also do not make use of the total number of reads (which by construction is the same between each condition). The only features are a one-hot encoding of the amino acids/nucleotides in the read. Specifically, as noted in the new Figure 2 caption:

More specifically, when applying MBE to the simulated data, we generate N training reads for condition A, and N training reads for condition B (i. e., equal class sizes). Then, we train a classifier on the N +N = 2N data points, where each data point is a one-hot encoding of one read sequence (one could add any other features as desired, such as a mapped genomic position) with a corresponding label (−1 for class A or +1 for class B). The same read can appear more than once, and may appear with discordant labels (e.g. if it appears in both conditions). The only difference in the implementation on the real data as compared to the simulated, is that the total number of reads may be quite different between the different conditions; to account for this, we re-weight the classifier loss function by the total number of reads in each condition to equalize the impact of each condition on the classifier (see Online Methods).

3) How can we distinguish the different fractions of lowest test sequences in sub- figures?, for example a, b, etc.

First, as now noted in the caption, “a 0.2-fraction of lowest test sequences corresponds to the sequences in the bottom 20% of all unique test set sequences after ranking by ground truth fitness. To produce each curve, we computed Spearman correlation for every fraction of lowest test sequences between 0.01 and 1.0, inclusive, in 0.01 increments for each method, although the legend shows dot sizes only in increments of 0.2 to reduce clutter.”

Second, each different fraction has a different-sized marker in the figure, as shown in the legend, although in retrospect we see that the legend could be confusing, suggesting only 5 dot sizes, whereas there were actually 100. Our intent was for this to showcase a continuum, with the dots blurring together to look like a blurred “swoosh” that conveys the overall trend. In light of your comment, we considered coloring the dots, but with the transparency used, this would not be useful. We also considered reducing to only 5 or 6 dot sizes in the plot, but as this would remove the continuum that is visible, we decided against it. However, if the reviewer has some specific suggestions on how we might change the plot to be more informative, we’d be happy to do so. (We note that with such figures in the Extended data, color is already used, and so cannot be used again for this kind of representation.)

Reviewer #2:

Major comments:

1. Given the various comparisons of models and datasets and made in this paper, it would be beneficial to have an overall schematic of the study design as well as visual representation of the proposed analysis approach as the first figure.

Indeed. We have now added to the revised manuscript new figures containing: a visualization of the different types of sequencing datasets (i.e. sparse reads, short reads, and hybrid reads) discussed throughout the manuscript (Fig. 1); a visualization of MBE (Fig. 2); and an overview the models and datasets used in the simulated study (Fig. 3).

2. I believe the paper would benefit from rearranging some text and sections to improve readability - for example, introducing datasets (e.g real experimental data) directly before the performance results to prevent the reader from jumping back and forth.

Thanks for this suggestion. In our revisions, we have consolidated all of the information regarding the setup of the experiments using the real experimental data into a single section, Overview of experiments with real data, which we have relocated so that it appears directly before Results on real experimental data. These revisions also have the effect of consolidating the overview of the simulation experiments with the simulated results, which we agree improves the overall readability of the Results.

3. Since the focus of this paper was to introduce a new analysis approach for other researchers to use, the code should be packaged as a library and/or should have clearly defined instructions for implementation, with practical examples

As this was also requested by Reviewer #1 we copy our response from there again here for convenience:

Agreed. We have now created a ‘model_based_enrichment’ Python package which has been uploaded with the revision, and will be publicly released upon acceptance. Our package provides a general-purpose implementation of MBE to be used on a dataset of sequences and corresponding sequencing counts, for two or more conditions. The package can readily use any classifier in scikit-learn [8], one of the most widely-used, and easy-to-use machine learning libraries for Python, for which we provide clear demonstrations. We also include an example of how one can go beyond scikit-learn; namely, we show how to use any TensorFlow classifier. In particular, we provide a clear example demonstrating how to use MBE with the specific model architectures appearing in our manuscript.

4. The real experimental datasets were all short-read based, are there any long- read or hybrid (long + short read) datasets that could also be included for confirmation of performance? Particularly since MBE showed the biggest performance advantage against wLER in these settings.

Unfortunately, despite a substantial literature search, we have been unable to identify any available long-read or hybrid-read datasets that include both (1) sequencing data from two conditions, and (2) a validation set of low-throughput experimental/biophysical measurements. Typically, publicly available datasets—such as [6-7]—include (a subset of) sequencing data, but no quantitative experimental measurement for validation. If the reviewer is aware of any available long-read datasets that meet both of the requirements (1) and (2), we would be delighted to incorporate it into the manuscript!

5. While the main motivation for this approach was to make enrichment predictions for unobserved sequences, the authors also touch on a more classical analysis of estimation of log-enrichment. For this second case, as I understood it, it was an alternative for count-based enrichment analysis, e.g. for RNA-seq. If this is correct, should this approach not be further assessed against popular negative binomial modelling methods, such as DEseq2 or edgeR? Alternatively, should the focus be reframed to only consider predictions for unobserved sequences?

One important issue that complicates the inclusion of such a comparison is that negative binomial modeling methods like DEseq2 and edgeR require that the data contain biological replicates, which are used to estimate the dispersion parameter [5]. As the foundation of our experiments was with no replicates (such as we typically have in selection experiments), this means that with our current simulation set-up, negative binomial methods cannot be compared to. The same is true for the real data experiments. However, because of the reviewer’s comments, we have now generated two more replicates for each of our simulated data sets, so that triplicates are available on which to use DEseq2. The replicates differ in the random seed that was used to sample read counts from the ground truth fitness. In our experiments, DEseq2 tends to be outperformed by count-based enrichment (Extended Data Fig. 4), despite having access to three times as much data (because of the difference in the amount of data, we put it in the Extended Data), possibly because it is expecting replicates of a different nature, although we are unsure. We welcome feedback from the reviewers on suitability of this experiment and plot for inclusion in the paper.

Minor comments:

1. Illumina and Pacbio sequencing error rates are estimated around 0.1%, not 1% (however, Nanopore long read error rates are around 1%). This should be corrected in the text.

We appreciate the reviewer catching this error, and we have corrected the value of the error rate in the revised manuscript.

2. Could the authors please re-order and group simulated experiments in figure 1 and extended data figure 3 in some logical way, perhaps following the order in table 1 (different length insertions, short vs long avGFP mutagenesis, AAV - different sparseness of long reads, short reads and hybrid experiment)?

Initially, we had ordered the results in these figures (Fig. 4 and Extended Data Fig. 1 in the revised manuscript) in decreasing order of the performance gap between MBE and wLER, thinking it may increase interpretability by highlighting the datasets that yielded the largest performance benefits. However, in light of the reviewers’ comments, we agree that the overall readability is improved by maintaining a consistent ordering of the datasets throughout the manuscript, and in our revisions we have reordered the results in the figures based on Table 1, which we agree is an improvement.

3. Could the authors please use consistent names for datasets in figure 4 as listed in table 2?

Yes, we can! We have revised this figure and its caption to use the library names as listed in Table 2.

4. In figure 4, alongside Spearman correlation, it would also be helpful to plot the individual correlations of predictions vs functional validation per sequence.

Indeed, it turns out that we already had such plots (Extended Data Fig. 12 in the revised manuscript), although we had failed to sufficiently highlight them in the original manuscript, and have now done so (line 1462, and Figure 7 caption).

References

1. Guo, C., Pleiss, G., Sun, Y., & Weinberger, K. Q. (2017, July). On calibration of modern neural networks. In International conference on machine learning (pp. 1321- 1330). PMLR.

2. Gneiting, T., Balabdaoui, F., & Raftery, A. E. (2007). Probabilistic forecasts, calibration and sharpness. Journal of the Royal Statistical Society Series B: Statistical Methodology, 69(2), 243-268.

3. Niculescu-Mizil, A., & Caruana, R. (2005, August). Predicting good probabilities with supervised learning. In Proceedings of the 22nd international conference on Machine learning (pp. 625-632).

4. Sugiyama, M., Suzuki, T., & Kanamori, T. (2012). Density-ratio matching under the Bregman divergence: a unified framework of density-ratio estimation. Annals of the Institute of Statistical Mathematics, 64, 1009-1044.

5. Yan, F., Powell, D. R., Curtis, D. J., & Wong, N. C. (2020). From reads to insight: a hitchhiker’s guide to ATAC-seq data analysis. Genome biology, 21, 1-16.

6. Kanwar, N., Blanco, C., Chen, I. A., & Seelig, B. (2021). PacBio sequencing output increased through uniform and directional fivefold concatenation. Scientific reports, 11(1), 18065.

7. Starr, T. N., Greaney, A. J., Stewart, C. M., Walls, A. C., Hannon, W. W., Veesler, D., & Bloom, J. D. (2022). Deep mutational scans for ACE2 binding, RBD expression, and antibody escape in the SARS-CoV-2 Omicron BA. 1 and BA. 2 receptor-binding domains. PLoS pathogens, 18(11), e1010951.

8. Buitinck, L., Louppe, G., Blondel, M., Pedregosa, F., Mueller, A., Grisel, O., ... & Varoquaux, G. (2013). API design for machine learning software: experiences from the scikit-learn project. arXiv preprint arXiv:1309.0238.

**2^nd^ round**

**Reviewer 1**

The authors have addressed most of my concerns.

**Reviewer 2**

Thanks to the authors for addressing my comments.
The only additional suggestion is to provide an example input file for the model_based_enrichment package (I'd suggest including one of the simulated datasets used in this study).

**Authors’ response**

Reviewer #2:

The only additional suggestion is to provide an example input file for the model_based_enrichment package (I'd suggest including one of the simulated datasets used in this study).

We have now included a new example input file in the MBE package for demonstration

purposes.
